# Supplementary material for: Assessing early detection ability through spatial arrangements in environmental surveillance for poliovirus: A simulation-based study
Source: PLoS One. 2025 Jul 9;20(7):e0325789. doi: 10.1371/journal.pone.0325789 (PMC12240349; doi:10.1371/journal.pone.0325789)
Supplement: S1 Text — (PDF) [file pone.0325789.s001.pdf]

Supplementary material for

Assessing early detection ability through spatial arrangements in environmental surveillance for poliovirus: a simulation-based study

Toshiaki R. Asakura*, Kathleen M. O'Reilly

* Corresponding to toshiaki.asakura1@lshtm.ac.uk

Contents

1 Supplementary Methods 3
1.1 Data 3
1.2 Ethical statement 5
1.3 Modelling framework 6
1.3.1 Transmission model 6
1.3.2 AFP surveillance model 10
1.3.3 Environmental surveillance model 11
1.3.4 Importation risk distributions 12
1.3.5 ES site layout strategies and a patch-level ES population coverage (p_c) 14
1.3.6 Model parameter specification 17
1.4 Outcome measures 19
1.5 Average minimum distance to ES-covered patches and simulated early detection probability. 19
2 Supplementary results 21
2.1 Characteristics for the top 20 populous patches 21
2.2 Simulation results under a single patch setting. 21
2.3 Different visualisations for the main analysis 24
2.4 Sensitivity analysis on the patch-level ES population coverage for different scenarios 26
3 References 28

List of Tables

Table S1. OPV coverage (%), HEXA coverage (%) and effective immunisation proportion (EIP, %) by districts in South Africa. 4
Table S2. Discrete Markov process for each time step. 9
Table S3. Approximated ES-covered population size in districts with ES sites in South Africa as of 27 November 2023. 15
Table S4. Observed and simulated district-level ES population coverage for the ES-POP varying the patch-level ES population coverages (p_c) given the simulated national ES population coverage was fixed at the observed one (11.3%). 16
Table S5. Model Parameters used for our simulation. 18
Table S6. Population size and effective immunisation proportion (EIP) for the top 20 populous patches. 21

List of Figures

Fig S1. Heatmap of population size in South Africa. (A) Children under 5 years old and (B) All ages. Patches with <100 children under 5 years old were removed from the analysis. 3
Fig S2. Estimated effective immunisation proportion by districts in South Africa, 2020 5
Fig S3. Schematic representation of our model, comprising of three parts: transmission model, AFP surveillance model and ES model. 6
Fig S4. Moving rates from origin *i* to destination *j* (π_{ij}) approximated by the radiation model for the three most populous patches. 8
Fig S5. The proportion of individuals excreting the poliovirus regardless of the amount of virus shedding over time, which was scaled to be one for the probability density function and the fitted probability density function. 10
Fig S6. Dose-response curve for the ES sensitivity parameter against the number of infectious individuals per 100,000 population in a single patch. 12
Fig S7. Importation risk distributions in a log10 scale (A) for IMP-POP, (B) IMP-AIR, and (C) IMP-LBC. 14
Fig S8. Environmental surveillance (ES) location maps. 17
Fig S9. Histogram of the effective reproduction number for each patch and corresponding outbreak probability with ≥10 infections. 20
Fig S10. Sensitivity analysis of parameters in a single patch setting. 23
Fig S11. Dose-response curves for estimated ES sensitivity parameters under different population sizes of children under 5 years old (N_c) in a single patch setting. 24
Fig S12. Proportion of each detection pattern (%) against national ES population coverage for six scenarios. 25
Fig S13. Proportion of each detection pattern (%) including the no detection pattern against the number of ES-covered patches for six scenarios. 25
Fig S14. Sensitivity analysis of the patch-level ES population coverage, p_c, for the ES-pop scenarios. 26
Fig S15. Sensitivity analysis of the patch-level ES population coverage, p_c, for the ES-LBC scenarios. 27

1 Supplementary Methods

1.1 Data

We collated the population size data from the WorldPop [1] at 100m spatial resolution for 5 bin age categories and created population size data for children under 5 years old and for all ages in South Africa. We aggregated the population data into a 20km spatial resolution (precisely 21.31km x 19.74km), resulting in 3,193 patches. We removed patches with less than 100 children under 5 years old and finally obtained 1502 patches (Fig S1). Through this process, we removed 0.61% population of children under 5 years old. We kept patch spatial resolution constant for all patches so that ES-covered areas in each patch were fixed. We applied the same procedure to create the population data for children under 5 years old in Mozambique at the same resolution.

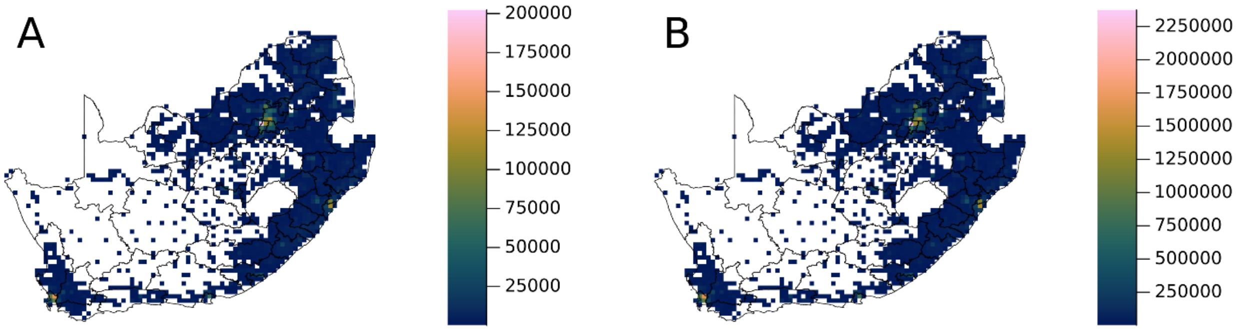

Fig S1. Heatmap of population size in South Africa. (A) Children under 5 years old and (B) All ages. Patches with <100 children under 5 years old were removed from the analysis. National and district borders for the maps were drawn with geoBoundaries under the CC-BY 4.0 license [2].

We collated the district-level vaccination coverage from the Expanded Programme on Immunisation National Coverage Survey Report 2020 in South Africa [3]. We summarised the coverage of oral polio vaccine (OPV) and hexavalent vaccine (HEXA) including inactivated polio vaccine (IPV) for each district in Table S1. We assumed those vaccinated with at least one IPV also completed two doses of OPV in our simulation, and classified children into immunised and unimmunised ones. Immunised children are assumed to have no susceptibility and transmissibility because of mucosal immunity induced by OPV and they are assumed not to develop AFP because of serum antibody induced by IPV, which allowed us to remove immunised children from the transmission dynamics. Since the last known polio case in South Africa as of May 2025 was documented in 1989 [4], immunity from natural infection was not considered.

We defined the proportion of children under 5 years old who were effectively immunised as the effective immunisation proportion (EIP) and calculated it considering district-level coverage of IPV and vaccine effectiveness against transmission. Let VE_p be the vaccine effectiveness per dose (0.63 against serotype 1 [5–7]) and the probability of immunisation after n doses of IPV (VE_n) is calculated as $1 - (1-VE_p)^n$. Considering the IPV coverage for nth dose ($C_{v,n}$), the EIP can be estimated considering VE_n and the IPV coverage for nth dose ($C_{v,n}$), which is described by $C_{v,4}VE_4 + (C_{v,3} - C_{v,4})VE_3 + (C_{v,2} - C_{v,3})VE_2 + (C_{v,1} - C_{v,2})VE_1$. When $C_{v,n} < C_{v,n+1}$, we assumed $C_{v,n}$ - $C_{v,n++1}$ to be zero. We calculated the district-level EIPs (Table S1 and Fig S2 in S1 text) and assigned them to the corresponding patches. The average EIP weighted by the population size of children under 5 years old in each patch in South Africa was 91.9%.

Table S1. OPV coverage (%), HEXA coverage (%) and effective immunisation proportion (EIP, %) by districts in South Africa.

District	Sample size	OPV0[a]	OPV1	HEXA1[b]	HEXA2	HEXA3	HEXA4	EIP[c]
West Coast	74	97.3	95.9	97.3	97.3	89.2	82.4	94.3
Cape Winelands	179	96.1	94.4	95.0	94.4	93.9	88.8	92.8
Overberg	143	97.2	94.4	94.4	94.4	96.5	84.6	94.3
Eden	121	96.7	88.4	95.9	95.0	90.9	89.3	93.3
Central Karoo	33	100.0	100.0	100.0	100.0	100.0	97.0	98.0
Namakwa	27	100.0	96.3	96.3	100.0	100.0	92.6	97.9
Pixley ka Seme	32	93.8	87.5	90.6	81.3	78.1	68.8	85.0
Z F Mgcawu	61	98.4	98.4	96.7	100.0	96.7	83.6	97.3
Frances Baard	200	99.0	98.0	99.0	99.5	98.5	90.5	97.3
Cacadu	348	97.1	95.4	96.0	96.3	94.8	87.6	94.1
Amathole	220	98.6	96.4	97.7	96.8	95.0	86.4	95.1
Chris Hani	79	98.7	92.4	92.4	97.5	93.7	87.3	95.0
Joe Gqabi	110	95.5	92.7	92.7	91.8	93.6	81.8	92.0
O.R.Tambo	372	95.4	90.3	86.6	87.9	84.4	76.3	85.6
Xhariep	81	97.5	95.1	96.3	92.6	93.8	76.5	93.8
Lejweleputswa	285	98.6	97.9	99.3	97.9	97.9	87.4	96.6
Thabo Mofutsanyane	50	100.0	96.0	98.0	98.0	98.0	86.0	95.8
Fezile Dabi	128	91.4	81.3	82.0	82.8	82.8	82.0	81.2
Ugu	187	97.3	96.8	97.9	98.4	96.3	88.8	96.1
Umgungundlovu	60	96.7	96.7	93.3	96.7	96.7	83.3	94.5
Uthukela	700	95.3	93.7	93.3	92.7	92.7	80.6	91.0
Umzinyathi	205	97.6	97.6	97.1	96.6	95.6	83.9	94.6
Amajuba	470	96.0	95.3	95.3	93.6	93.8	83.2	92.8
Zululand	399	96.5	95.5	95.0	95.2	93.7	77.9	92.7
Umkhanyakude	243	97.1	95.5	93.8	94.2	93.8	87.2	92.2
Uthungulu	986	92.4	92.3	92.7	91.7	91.3	82.8	90.3
iLembe	359	79.9	78.6	76.9	76.3	76.6	72.4	75.4
Gert Sibande	284	99.3	98.2	98.2	98.2	97.9	84.9	95.9
Nkangala	328	90.2	87.5	88.7	88.4	86.9	78.0	86.5
Ehlanzeni	396	93.2	92.9	89.9	89.9	89.6	81.1	87.9
Mopani	680	93.5	91.5	91.9	92.1	90.4	81.6	89.9
Vhembe	589	93.0	92.5	92.2	91.9	90.8	82.0	90.0
Capricorn	493	87.4	85.8	86.0	86.6	85.8	71.6	84.4
Waterberg	111	96.4	95.5	96.4	94.6	92.8	73.9	93.1
Bojanala	299	94.3	91.6	93.0	91.3	90.6	83.3	90.3
Ngaka Modiri Molema	484	97.1	96.7	94.8	95.2	93.2	82.0	92.8
Dr Ruth Segomotsi Mompati	106	97.2	97.2	97.2	95.3	95.3	84.9	94.4
Dr Kenneth Kaunda	210	95.7	94.8	94.8	93.3	91.9	83.8	92.1
Sedibeng	514	94.6	92.6	94.2	93.8	93.0	84.0	91.9
Sisonke	597	96.0	95.1	94.6	94.1	94.5	87.1	92.8
Alfred Nzo	271	98.2	95.9	96.7	96.7	95.6	83.4	94.4

John Taolo Gaetsewe	5	100.0	100.0	100.0	100.0	100.0	100.0	98.1
Sekhukhune	345	92.8	89.6	91.6	91.9	90.7	82.6	89.8
West Rand	382	97.4	96.9	97.4	96.1	95.8	90.1	94.9
Buffalo City	211	98.6	95.3	95.3	95.3	94.8	86.3	93.2
City of Cape Town	101	97.0	98.0	97.0	97.0	94.1	90.1	94.7
Ekurhuleni	890	97.1	96.1	96.6	95.5	94.9	86.9	94.1
eThekwini	887	94.0	92.6	92.4	91.7	91.8	83.8	90.3
City of Johannesburg	1710	97.0	96.4	95.6	95.3	94.8	87.8	93.4
Mangaung	187	98.9	97.9	97.3	98.4	97.9	87.7	96.2
Nelson Mandela Bay	326	94.5	93.3	93.9	92.9	92.0	86.5	91.5
City of Tshwane	621	98.9	96.3	96.5	95.3	95.8	86.5	94.5

[a] OPV: Oral polio vaccine
[b] HXA: Hexavalent vaccine including diphtheria, tetanus, acellular pertussis, Haemophilus influenzae type b, inactivated polio vaccine (IPV), and hepatitis B vaccines.
[c] EIP: Effective immunisation proportion.

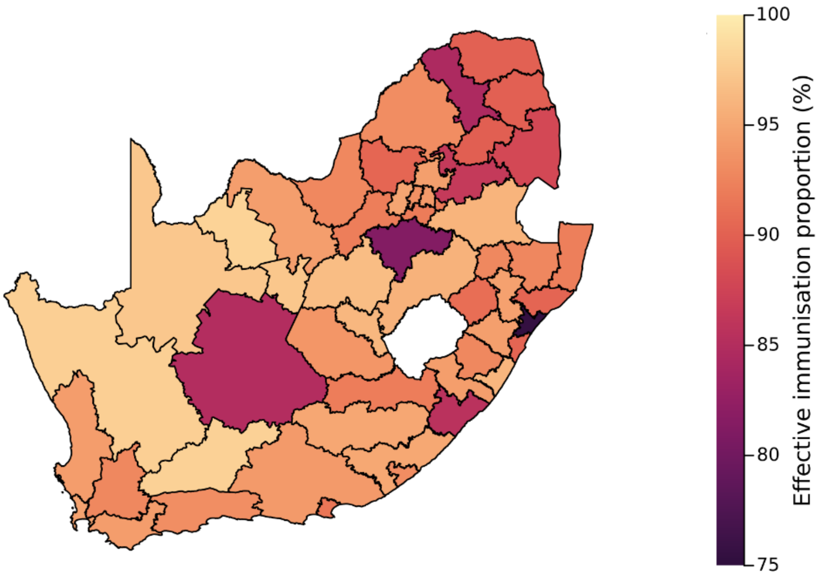

Fig S2. Estimated effective immunisation proportion by districts in South Africa, 2020. National and district borders for the maps were drawn with geoBoundaries under the CC-BY 4.0 license [2].

1.2 Ethics statement

This study was assessed by the Research Governance & Integrity Office at the London School of Hygiene & Tropical Medicine as not requiring ethical approval due to its study design as a secondary data analysis on 9 May 2023 (MSc ethics reference number: 29004). The location and population size covered by each wastewater plant were provided by the National Institute of Communicable Diseases (NICD) in South Africa on 18th March 2024. All the other data are publicly available.

1.3 Modelling framework

To assess the early detection ability of environmental surveillance (ES) over acute flaccid paralysis (AFP) surveillance, we constructed the stochastic spatiotemporal model among unimmunised children under 5 years old considering the case-detection process through the AFP surveillance and ES (Fig S3). We utilised South Africa as a case study of a non-endemic country and assumed a single introduction of a patient with wild poliovirus serotype 1 (WPV1). Following other modelling studies on polio [6,8,9], our model comprises three components: transmission model, AFP surveillance model and ES model (Fig S3). We used the detection patterns and first detection timing by ES and AFP surveillance as primary outcomes to quantify the early detection ability while we varied the number of ES-covered patches. We prepared three importation risk distributions and two ES site layout strategies, totalling six scenarios, to account for limited knowledge of importation risks (Fig 1).

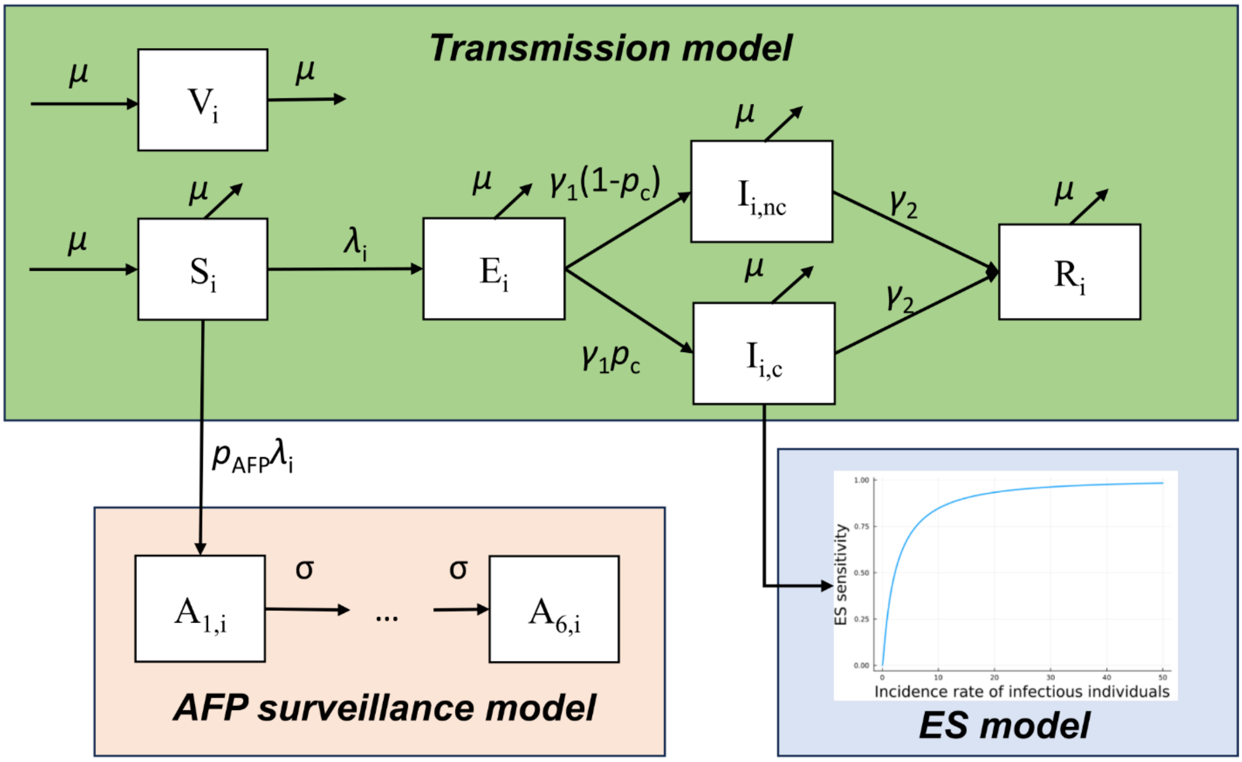

Fig S3. Schematic representation of our model, comprising of three parts: transmission model, AFP surveillance model and ES model. Each compartment is defined as follows: S, susceptible population; E, latent population; I, infectious population; R, recovered population; V, immunised population; and A_1 through A6, population who are developing AFP. ES sensitivity is dependent on the incidence rate of infectious polio cases. The subscript of *i* corresponds to the location. Abbreviations: ES, environmental surveillance; AFP, acute flaccid paralysis.

1.3.1 Transmission model

We employed the stochastic spatiotemporal SEIR model to describe the transmission dynamics. The SEIR model simulates the process in which susceptible individuals (S) get infected, passing through a latent period (E) and infectious period (I), and then recover from infection (R). We only considered unimmunised children under 5 years old since most reported patients with AFP were

within this age group [10]. We assumed no waning of immunity from vaccination or natural infection. Considering our simulation length (i.e. 3 years), we introduced the birth and removal rate from this age cohort (μ) to reflect the population dynamics. We classified infectious compartments into the one covered by the ES (I_c) and the one not covered by the ES (I_{nc}) but we assumed no difference in infectiousness between the two compartments.

The force of infection at patch *i* at day *t* ($\lambda_{i,t}$) corresponds to the rate at which susceptible individuals at patch *i* at day *t* get infected and is described by

$$\lambda_{i,t} = \frac{\beta}{N_{i,c}}\left[(1-\alpha)(I_{i,c,t} + I_{i,nc,t}) + \alpha \sum_{j \neq i} \pi_{ji}(I_{j,c,t} + I_{j,nc,t})\right], \tag{S1}$$

where β corresponds to the transmission rate, and α corresponds to the travelling rate between patches, at which individuals are outgoing from the original patch. The population aged under 5 years old at patch *i* is denoted as $N_{i,c}$. It is noted that immunised populations (V_i) contributed to herd immunity and its population size was given by $N_{i,c}$ multiplied by the EIP at patch *i* (EIP_i). Then, the initial susceptible population at patch *i* (S_i) was given by $N_{i,c}$ ($1-EIP_i$). We related the basic reproduction number (R_0) and β through the equation $R_0 = \beta/\gamma_2$ where $1/\gamma_2$ corresponds to an infectious period.

The second term of Equation S1 corresponds to the hazard from other patches, considering the travelling rate (α) and moving rates of travellers from origin *j* to destination *i* among all travellers moving from patch *j* (π_{ji}). We adopted the frequency-dependent model for between-patch transmission, which was theoretically investigated [11,12], while another modelling study on poliovirus employed density-dependent transmission [6]. We used the radiation model to approximate moving rates by

$$\pi_{ij} \propto \frac{N_{i,c}N_{j,c}}{(N_{i,c} + N_{ij,c}^{s})(N_{i,c} + N_{j,c} + N_{ij,c}^{s})}, \tag{S2}$$

which is scaled to one for each origin. We denote $N_{ij,c}^{s}$ as the aggregated population aged under 5 years old within the circular area with a radius extending from patch *i* to patch *j*, centred at patch *i* but excluding the source and destination population. The histogram and heatmap of moving rates (π_{ij}) of the top 3 populous patches are shown in Fig S4. The previous study suggested in the context of polio disease, the radiation model not only outperformed the gravity model [13–15], but also excelled when compared to the gravity model calibrated to mobile phone data [13].

We adopted the discrete Markov process with daily time steps for our stochastic simulations. Transition events, effects and sampling ways of new states were summarised in Table S2.

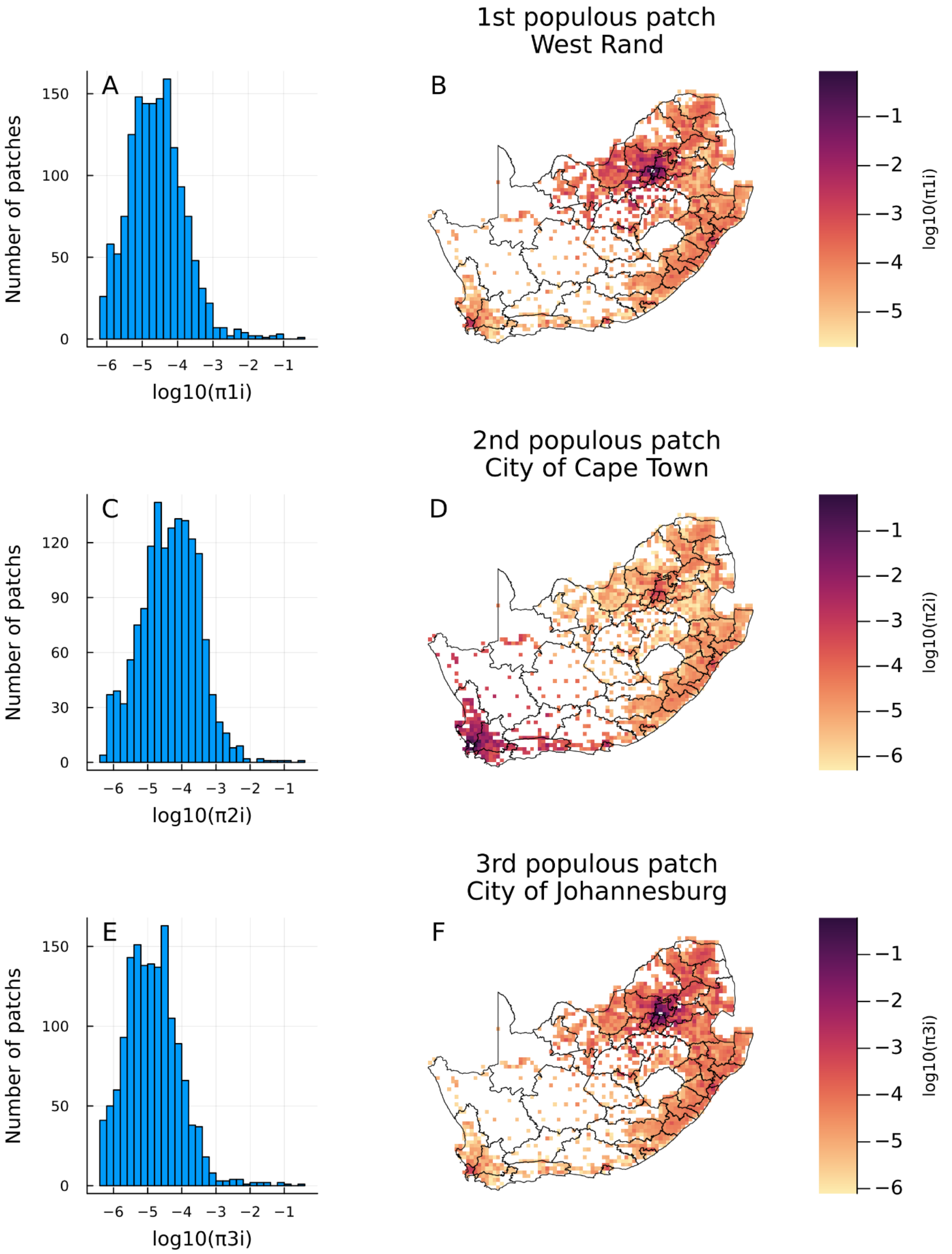

Fig S4. Moving rates from origin *i* to destination *j* (π_{ij}) approximated by the radiation model for the three most populous patches. (A, C, E) Histogram of π_{ij} and (B, D, E) heatmap of π_{ij} with colour bar values on a log10 scale for (A, B) West Rand, (C, D) City of Cape Town and (E, F) City of Johannesburg.

Table S2. Discrete Markov process for each time step.

Event (patch i)	Effect	Sampling way
Birth of susceptible individuals	$(S_{i,t+1}) \leftarrow (S_{i,t} + Z_{i,t}^{(N_{i,u})})$	$Z_{i,t}^{(N_{i,u})} \sim \text{Binomial}(N_{i,u}, 1 - e^{-\mu})$
Removal of susceptible individuals because of age eligibility	$(S_{i,t+1}) \leftarrow (S_i - Z_{i,t}^{(S_i)})$	$Z_{i,t}^{(S_{i,t})} \sim \text{Binomial}(S_{i.t}, 1 - e^{-\mu})$
Infection of susceptible individuals	$(S_{i,t+1}, E_{i,t+1}) \leftarrow (S_{i,t} - Z_{i,t}^{(S_{i.t}, E_{i,t})}, E_{i,t} + Z_{i,t}^{(S_{i,t}, E_{i,t})})$	$Z_{i,t}^{(S_{i,t}, E_{i,t})} \sim \text{Binomial}(S_{i,t}, 1 - e^{-\lambda_{i,t}})$
New individuals who will develop AFP	$(A_{1,i,t+1}) \leftarrow (A_{1,i,t} + Z_{i,t}^{(A_{1,i,t})})$	$Z_{i,t}^{(A_{1,i,t})} \sim \text{Binomial}(Z_{i,t}^{(S_{i,t}, E_{i,t})}, p_{AFP})$
Removal of latent individuals because of age eligibility	$(E_{i,t+1}) \leftarrow (E_{i,t} - Z_{i,t}^{(E_{i,t})})$	$Z_{i,t}^{(E_{i,t})} \sim \text{Binomial}(E_{i,t}, 1 - e^{-\mu})$
Becoming infectious	$(E_{i,t+1}, I_{i,t+1}) \leftarrow (E_{i,t} - Z_{i,t}^{(E_{i,t}, I_{i,t})}, I_{i,t} + Z_{i,t}^{(E_{i,t}, I_{i,t})})$	$Z_{i,t}^{(E_{i,t}, I_{i,t})} \sim \text{Binomial}(E_{i,t}, 1 - e^{-\gamma_1})$
Removal of infected individuals because of age eligibility	$(I_{i,t+1}) \leftarrow (I_{i,t} - Z_{i,t}^{(I_{i,t})})$	$Z_{i,t}^{(I_{i,t})} \sim \text{Binomial}(I_{i,t}, 1 - e^{-\mu})$
Recovery from infection	$(I_{i,t+1}, R_{i,t+1}) \leftarrow (I_{i,t} - Z_{i,t}^{(I_{i,t}, R_{i,t})}, R_{i,t} + Z_{i,t}^{(I_{i,t}, R_{i,t})})$	$Z_{i,t}^{(I_{i,t}, R_{i,t})} \sim \text{Binomial}(I_{i,t}, 1 - e^{-\gamma_2})$
Removal of recovered individuals because of age eligibility	$(R_{i,t+1}) \leftarrow (R_{i,t} - Z_{i,t}^{(R_{i,t})})$	$Z_{i,t}^{(R_{i,t})} \sim \text{Binomial}(R_{i,t}, 1 - e^{-\mu})$
Progression of the incubation period of AFP from stage k to k +1	$(A_{k,i,t+1}, A_{k+1,i,t+1}) \leftarrow (A_{k,i,t} - Z_{i,t}^{(A_{k,i,t}, A_{k+1,i,t})}, A_{k+1,i,t} + Z_{i,t}^{(A_{k,i,t}, A_{k+1,i,t})})$	$Z_{i,t}^{(A_{k,i,t}, A_{k+1,i,t})} \sim \text{Binomial}(A_{k,i,t}, 1 - e^{-\sigma})$

[a] $N_{i,u}$ denotes unvaccinated individuals at patch i, calculated as the product of population aged under 5 years old and effective immunisation proportion at patch i.

We estimated the infectious period ($1/\gamma_2$) using the proportion of infected individuals shedding any amount of virus over time, which was provided by expert opinions [16]. Formally, the time course of infectiousness can be obtained by the product of the proportion of individuals shedding any amount of virus and non-linear transformation of the amount of virus shedding given a person excretes the virus each day since infection [16]. Since we do not know the functional form for the latter data, we regarded the first one as the infectiousness for simplicity.

Since the compartment model implicitly assumes the transition process from one compartment to another follows the exponential distribution, the distribution of infectiousness over time can be described as the convolution of two exponential distributions:

$$f(t) = \int_0^s \gamma_1 e^{-\gamma_1(t-s)} \gamma_2 e^{-\gamma_2 s} ds = \frac{\gamma_1\gamma_2}{\gamma_2 - \gamma_1}(\mathrm{e}^{-\gamma_1 t} - \mathrm{e}^{-\gamma_2 t}), \tag{S3}$$

where the reciprocal, $1/\gamma_1$, corresponds to the mean latent period and is assumed to be 4 days, and $1/\gamma_2$ corresponds to the mean infectious period. We minimised the Kullback-Leibler divergence between the observed distribution and *f(t)*, estimating $1/\gamma_2$ to be 15.02 days. The fitted curve is shown in Fig S5.

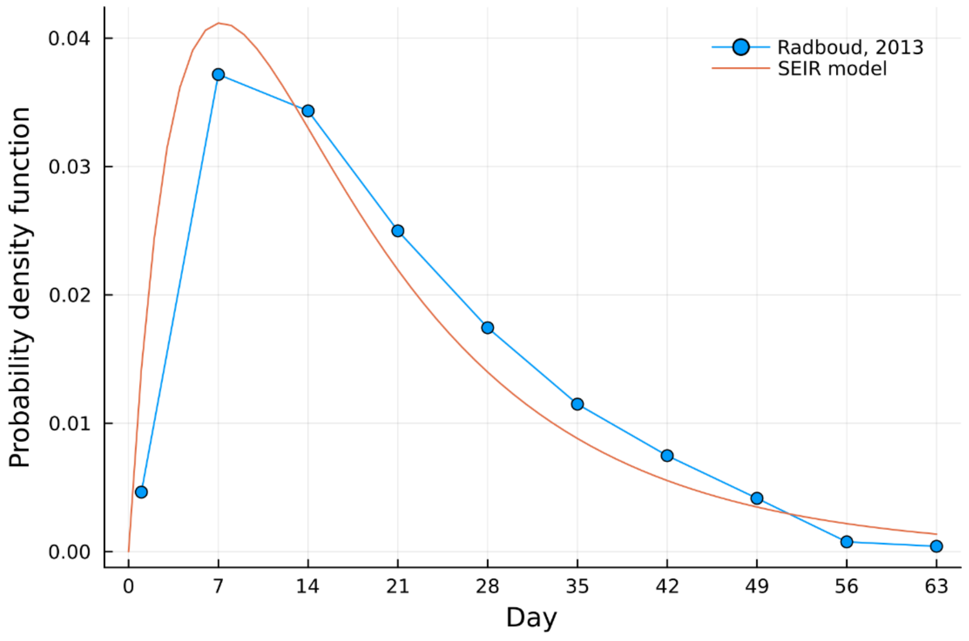

Fig S5. The proportion of individuals excreting the poliovirus regardless of the amount of virus shedding over time, which was scaled to be one for the probability density function and the fitted probability density function.

1.3.2 AFP surveillance model

We assumed the probability of developing AFP given infection with poliovirus to be 1/200, which was denoted as p_{AFP}. Patients developing AFP are correctly reported if they successfully undergo the following process: seeking healthcare, being tested, and getting a positive result. We denoted P_H, $P_{AFP, sample}$, and $P_{AFP ,test}$ as the probability of each process. Then, the number of reported patients with AFP at patch *i* at time *t*, $O_{i,t}$, was described as

$$O_{i,t} \sim \mathrm{Binomial}\left(Z_{i,t}^{(A_{5,i,t}, A_{6,i,t})}, P_H P_{AFP,sample} P_{AFP,test}\right), \tag{S4}$$

where the number of individuals who newly develop AFP at patch *i* at day *t* is denoted as $Z_{i,t}^{(A_{5,i,t}, A_{6,i,t})}$.

The incubation period for developing AFP was assumed to be 16.5 days [17,18] and we prepared six compartments with transition rates of 0.329 days^{-1} to be aligned with the incubation period distribution [6]. The number of compartments and transition rates were estimated in the previous study [6] by fitting an Erlang distribution to 36 independent data intervals from poliovirus exposure to the onset of AFP. In the present study, we did not consider a delay in medical consultations, conducting tests and notifications to authorities. These processes usually take time [19] and should be accounted for when results are interpreted.

1.3.3 Environmental surveillance model

There are limited empirical data to compare the incidence of poliovirus against the probability of a positive ES sample, which is a required parameter in this analysis. Instead, we assumed that ES sensitivity for poliovirus was comparable to that observed for COVID-19, and estimated the required parameters using the wastewater surveillance data in the US, published by Wu et al. [20]. The data for COVID-19 is more amenable to analysis because of the intensity of clinical testing for SARS-CoV-2 and the (comparatively) low proportion of asymptomatic infections. To ensure the consistent quality of ES in each sampling site for estimation, we used sampling sites with both positive and negative samples, where daily newly reported cases on the day with positive samples were consistently higher than on the day with negative samples. Finally, 27 out of 353 sampling sites were eligible for the estimation.

We employed the log-normal distribution to estimate the dose-response curve for the ES sensitivity against the incidence rate of COVID-19 (thus also of poliovirus infection). Let $x_{i,t}$ be the number of newly reported COVID-19 cases at sampling site *i* on day *t*, $y_{i,t}$ be the indicator variable for an observed positive sample at sampling site *i* on day *t,* and $p_{i,t}$ be the probability of detecting a positive sample at site *i* on day *t*. We minimized the following likelihood function to estimate the parameters of the lognormal distribution (μ_{ES} and σ_{ES}):

$$L = \prod_i \prod_t p_{i,t}^{y_{i,t}} \left(1 - p_{i,t}\right)^{1-y_{i,t}}, \qquad \text{(S5)}$$

$$p_{i,t} = G\left(100{,}000 \frac{x_{i,t}}{N_{i,FW}}; \mu_{ES}, \sigma_{ES}\right) = \Phi\left(\frac{\log\left(100{,}00 \frac{x_{i,t}}{N_{i,FW}}\right) - \mu_{ES}}{\sigma_{ES}}\right)$$

where *G* denotes the cumulative density function of the log-normal distribution, $N_{i,FW}$ denotes the population in the state with sampling site *i,* which was given in Fuqing Wu et al. [20], and Φ corresponds to the cumulative density function of the standard normal distribution. We obtained 0.818 for μ_{ES} and 1.45 for σ_{ES}.

The estimated parameters here will be inappropriate in terms of differences in the amount and length of virus shedding between SARS-CoV-2 and poliovirus in addition to differences in diagnostics for the detection of virus. In the main text, we conducted the sensitivity analysis ranging the ES sensitivity from 10 times higher to 10 times lower. We multiplied 1/k for previous data to obtain parameters for k times higher ES sensitivity. The dose-response curves for those ES sensitivity parameters are shown in Fig S6. σ_{ES} was estimated to be constant across different ES sensitivity assumptions and μ_{ES} was estimated to be 3.121, 1.917, -0.281 and -1.485 for 10 times lower, 3 times lower, 3 times higher, and 10 times higher ES sensitivity, respectively.

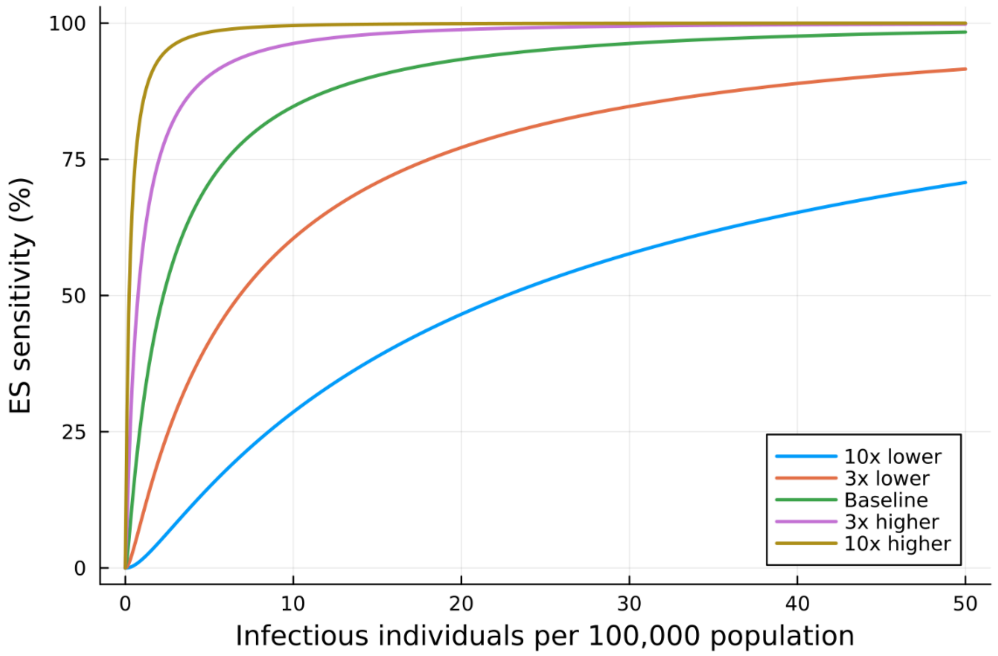

Fig S6. Dose-response curve for the ES sensitivity parameter against the number of infectious individuals per 100,000 population in a single patch.

We modelled the case-detection process through ES as the binomial process in our simulation study. Let $n_{i,t}$ be the indicator variable for conducting environmental sampling at patch *i* at day *t* (e.g. given monthly sampling, $n_{i,t}$ takes one on a single day, and takes zero for the remaining days within a month in ES-covered patches while $n_{i,t}$ always takes zero in non-ES-covered patches), and $P_{ES,\ test}$ be the test sensitivity given a collected sample contains a detectable level of virus. The number of polio-positive samples in wastewater ($w_{i,t}$) is given by

$$w_{i,t} \sim \text{Binomial}\left(n_{i,t}, G\left(100{,}000\frac{I_{i,c,t}}{N_i p_c}; \mu_{ES}, \sigma_{ES}\right) P_{ES,test}\right). \tag{S6}$$

It is noted that in each patch, p_c% of the population was assumed to be covered by the ES, and we separately considered the infectious individuals covered by ES ($I_{i,c,t}$) or not ($I_{i,nc,t}$) in the compartment model. Here N_i denotes the population of all ages at patch *i* (not limited to children under 5 years old).

1.3.4 Importation risk distributions

Importation risk at patch *i* refers to the probability that WPV1 is introduced to that specific patch and importation risk distribution refers to the distribution of importation risk across patches. We considered three importation risk distributions (Fig S7), which are denoted as IMP-POP, IMP-AIR, and IMP-LBC.

The IMP-POP refers to the 'Population size'-based importation risk distribution and assumes importation risk is proportional to the population size of each patch. Let r_i be the importation risk at patch *I* for the IMP-POP scenario and be described as

$$r_i = \frac{N_{i,c}}{\sum_i N_{i,c}}, \tag{S7}$$

where $N_{i,c}$ denotes the population size of children aged under 5 years old at patch *i*.

The IMP-AIR refers to the ‘International airport’-based importation risk distribution and assumes importation risk is proportional to international inbound travel volume in 2019 and further considers mobilisation from airports, which was approximated by the radiation model given in Equation S2. Without considering mobilisation from airports, importation risks would be too confined to a few patches, which are sometimes very rural areas and we did not think that importation risk distribution is a realistic one. South Africa holds three international airports and their international inbound travel volumes in 2019 were 4,342,611 for O.R. Tambo International Airport, 1,156,996 for Cape Town International Airport, and 188,243 for King Shaka International Airport (Fig 1D).

We denote l_1, l_2 and l_3 as the location of a patch with O.R. Tambo International Airport, Cape Town International Airport, and King Shaka International Airport, respectively. Let w_{li} be the proportion of the inbound travel volume at patch l_i with international airport *i*. The importation risk at patch *i* for the IMP-AIR is described as the average of moving rates from three international airports weighted by their inbound travel volumes:

$$r_i = w_{l1}\pi_{(l1)i} + w_{l2}\pi_{(l2)i} + w_{l3}\pi_{(l3)i}. \tag{S8}$$

The IMP-LBC refers to the ‘Land border crossing’-based importation risk distribution and assumes importation risk is proportional to travelling volume from Mozambique. Here, we assumed poliovirus was circulating in Mozambique and importation from Mozambique to South Africa happened via land border crossing. Since mobilisation volume data between Mozambique and South Africa is not available, we approximated those mobilisations with the radiation model.

The probability of the presence of poliovirus in patch *j* in Mozambique was assumed to be proportional to the population size of children under 5 years old, and the movement from patch *j* in Mozambique to patch *i* in South Africa is given by the radiation model between the two countries. The importation risk at patch *i* in South Africa for the IMP-LBC is described as

$$r_i = \frac{\sum_{j\in S_{MOZ}} \alpha_M \pi_{ji} N_{j,c}}{\sum_{k\in S_{ZAF}} \sum_{j\in S_{MOZ}} \alpha_M \pi_{jk} N_{k,c}} = \frac{\sum_{j\in S_{MOZ}} \pi_{ji} N_{j,c}}{\sum_{k\in S_{ZAF}} \sum_{j\in S_{MOZ}} \pi_{jk} N_{k,c}}, \tag{S9}$$

where S_{MOZ} and S_{ZAF} represent the set of patches in Mozambique and South Africa, respectively. The travelling rate from Mozambique to South Africa (α_M) would be different from the travelling rate between patches in South Africa (α) but is cancelled out in Equation S9.

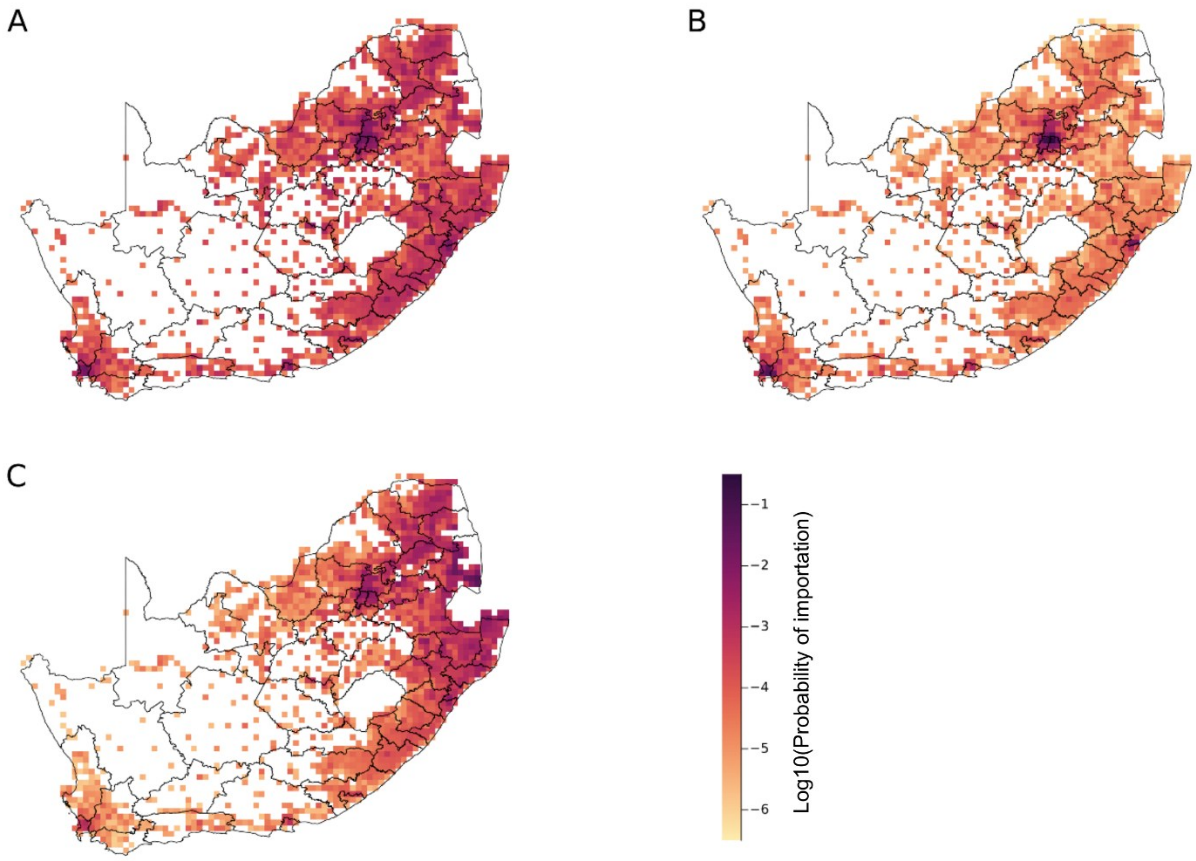

Fig S7. Importation risk distributions in a log10 scale (A) for IMP-POP, (B) IMP-AIR, and (C) IMP-LBC. National and district borders for the maps were drawn with geoBoundaries under the CC-BY 4.0 license [2].

1.3.5 ES site layout strategies and a patch-level ES population coverage (p_c)

ES site layout strategy determines a sequence of which patches are covered by the ES when we increase the number of ES-covered patches. We considered two ES site layout strategies: ES-POP and ES-LBC. The ES-POP refers to the ‘Population size’-based ES site layout strategy, and assumes the ES is implemented in the descending order of population size of each path. Hence, this strategy can cover the largest population size by the ES given the same number of ES-covered patches.

The ES-LBC refers to the ‘Land border crossing importation risk’-based ES site layout strategy, and assumes the ES is first implemented in a patch with a high importation risk via land border crossing from Mozambique. The importation risk from Mozambique is given as the same in Equation S9. The motivation to prepare the ES-LBC is to quantify the effectiveness of the strategic positioning of the ES sites against the high importation risks in rural areas. The animations of the incremental ES implementation from a single patch to all patches were prepared for both ES-POP and ES-LBC in gif-files (S1-2 Video). It is noted that the maximum national ES population coverage was set at 25% since we assumed the ES-population coverage in each patch (called a patch-level ES population coverage, p_c) to be 25%, which reason is explained below.

We aimed for our simulated ES site layout under the ES-POP to be closely aligned with the observed one. The ES site is generally prioritised to be placed in populous areas in the real-world setting. To quantify the similarity between simulated and observed ES layouts in South Africa, we first calculated the observed national and district-level ES population coverage (Table S3). Then, we varied a patch-level ES population coverage (p_c) fixing the simulated national ES population coverage to be the same as the observed one, and descriptively compared the simulated number of districts with ES sites and the corresponding district-level ES population coverage with the observed ones (Table S4).

We calculated the observed national and district-level ES population coverage using the location and ES-covered population of each wastewater plant, which was provided by the National Institute of Communicable Diseases in South Africa as of 18th March 2024. The ES-covered population was missing for Rooiwai Eastern and Daspoort wastewater plants and we imputed the median ES-covered population size of 350,000. We finally estimated the observed national ES population coverage in South Africa to be 11.3% (Table S3). The median and mean of the district-level ES population coverage among districts with ES sites were 30.6% and 22.5%, respectively.

We simulated the ES layout varying the patch-level ES population coverage (p_c) while fixing the simulated national ES population coverage to be the observed one of 11.3% (Table S4). When we set p_c at 100%, the ES sites were too concentrated and simulated district-level ES population coverages were much higher than observed ones. We chose p_c to be 25% for the main analysis considering the dispersion of ES sites and district-level ES population coverages (Table S4 and Fig S8).

Table S3. Approximated ES-covered population size in districts with ES sites in South Africa as of 27 November 2023.

Province	District	District-level population size	Wastewater plant name	ES-covered population size	District-level ES population coverage (%)
Eastern Cape	Buffalo City	760840	East Bank	141000	22.5
			Gonubie	30400	
Free State	Mangaung	826621	Bloemspruit	350000	66.5
			Sterkwater	200000	
Gauteng	City of Johannesburg	5540727	Northern	1200000	46.0
			Goudkoppies	500000	
			Bushkoppies	850000	
	City of Tshwane	3627986	Rooiwal Eastern	NA	NA
			Daspoort	NA	
	Ekurhuleni	3739653	Hartebeesfontein	100000	10.7
			Olifantsfontein	100000	
			Vlakplaats	200000	
KwaZulu Natal	eThekwini	3587907	Central	350000	18.6
			Northern	316425	
North West	Bojanala	1786678	Rustenburg	509000	28.5
Western Cape	City of Cape Town	4436413	Borcherd's Quarry	380000	18.9
			Zandvliet	460000	

Table S4. Observed and simulated district-level ES population coverage for the ES-POP varying the patch-level ES population coverages (p_c) given the simulated national ES population coverage was fixed at the observed one (11.3%).

Province	District	Observed district-level ES population coverage (%)	Simulated district-level ES population coverage (%)		
			p_c=1.0[a]	p_c=0.5[a]	p_c=0.25[a]
Eastern Cape	Buffalo City	22.5			19.6
	Nelson Mandela Bay	0.0		36.0	18.0
Free State	Mangaung	66.5			15.2
	Thabo Mofutsanyane	0.0			9.7
Gauteng	Ekurhuleni	10.7		17.5	25.0
	City of Johannesburg	46.0	37.1	50.0	25.0
	City of Tshwane	19.3		12.4	21.2
	West Rand	0.0	56.8	28.4	23.6
	Sedibeng	0.0			17.5
Kwazulu Natal	eThekwini	18.6	64.2	32.1	22.2
	iLembe	0.0		31.8	19.3
	Umgungundlovu	0.0			8.8
	Amajuba	0.0			9.1
Western Cape	City of Cape Town	18.9	36.7	40.7	22.8
	Cape Winelands	0.0			12.5
Limpopo	Waterberg	0.0			8.6
	Capricorn	0.0			7.9
Mpumalanga	Ehlanzeni	0.0			7.0
North West	Bojanala	28.5			11.6
Northern Cape	NA	NA			

[a] Blank in those columns represents no ES-covered patches.

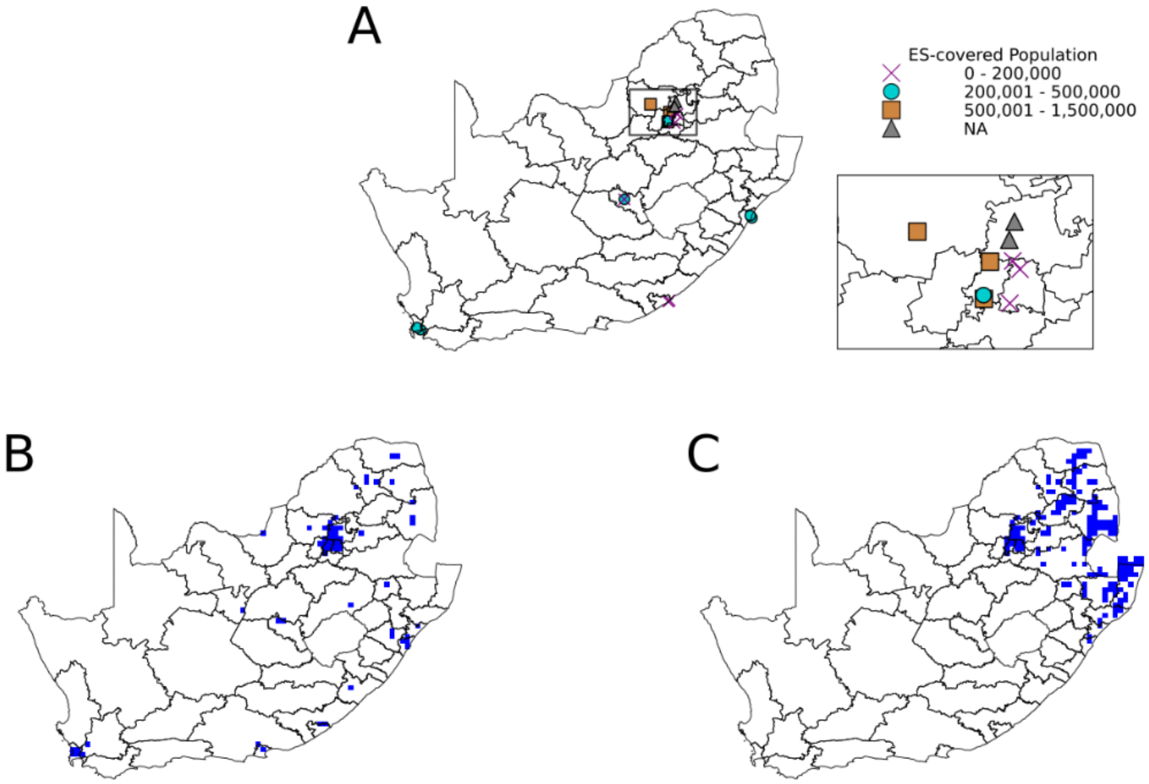

Fig S8. Environmental surveillance (ES) location maps. (A) Observed 17 ES sites in South Africa. Data points represent the location of observed ES sites, with each marker style indicating a specific range of ES-covered population sizes. The inset displays the expanded view of the areas with a high density of ES sites. (B, C) Simulated ES site layout when the simulated national ES population coverage was matched with the observed coverage given the patch-level ES population coverage was set at 25% (B) for ES-POP and (C) for ES-LBC. Blue squared areas represent patches with simulated ES sites. The number of ES-covered patches was 58 for ES-POP and 154 for ES-LBC. National and district borders for the maps were drawn with geoBoundaries under the CC-BY 4.0 license [2].

1.3.6 Model parameter specification

Parameter values used in our simulations are summarised in Table S5. We explained the rationale for the choice of several parameters.

We defined the effective reproduction number in the initial state at patch *i* as $R_{e,i}$, which represented the reproduction number for imported cases. The value $R_{e,i}$ is calculated by the product of R_0 and 1-EIP_i. We chose the basic reproduction number (R_0) to be 14, which resulted in 1.13, a slightly over one, for the average $R_{e,i}$ weighted by the population size of each patch. Since previous studies estimated R_0 for WPV1 around 5-10 [21,22], and other modelling studies assumed R_0 of 7.5-8.0 [23,24], we covered R_0 between 8 to 16 in the sensitivity analysis.

Regarding the AFP surveillance-related parameters, the stool adequacy rate refers to two stool specimens of sufficient quantity for laboratory analysis, collected at least 24 hours apart, within 14 days after the onset of paralysis. The stool adequacy rate in South Africa was reported 53% from 2016 to 2019 [25]. The procedures of handling samples from polio patients in the laboratory in South Africa followed the WHO Polio Laboratory Manual and supplement [25] and its sensitivity of polio detection was reported to be 0.97 [26].

We assumed monthly sampling (every 30 days) from the ES site in each patch for our simulation. Most African countries use a grab method to collect samples from wastewater and the sampling frequency is often 30 days due to manual labour burdens [27]. Some countries adopted a composite method for the ES, allowing daily or weekly sampling. GPEI recommends daily or weekly sampling but also accepts monthly sampling [28].

Table S5. Model Parameters used for our simulation.

**Parameter description**	**Values**	**Reference**
Per-dose vaccine effectiveness for IPV assessed by seroconversion	0.63	[29–31]
Average effective immune proportion (EIP) weighted by population size of children under 5 years old	91.9%	Estimated from the population data (WorldPop) and vaccine coverage data
Basic reproduction number, R_0	14	Assumed [21,22]
Average effective reproduction number in the initial state ($R_{e,i}$) at a national level	1.13	Estimated
Latent period, $1/\gamma_1$	4 days	[32]
Infectious period, $1/\gamma_2$	15.02 days	Estimated [16]
Transmission rate, β	0.93	Calculated by $R_0\,\gamma_2$
Travelling rate, α	0.05	Assumed [33]
Age criteria for population	Less than 5 years old	Assumed
Birth and removal rate, μ	1/(365*5)	Assumed
Transition rate of the incubation period for 6 compartments, σ	1/3.04 days^{-1}	Estimated [6,17,18]
Patch-level ES population coverage, p_c	0.25	Assumed
Paralysis-to-infection ratio, p_{AFP}	1/200	[34]
Probability of seeking healthcare, P_H	0.9	Assumed
Probability of stool sampling when a patient visits a hospital, $P_{AFP,\ sample}$	0.53	[25,35]
Sensitivity of poliovirus detection when stool is tested, $P_{AFP,\ test}$	0.97	[26,36]
Sensitivity of poliovirus testing of samples from sewage water, $P_{ES,\ test}$	0.97	[26,36]
ES sensitivity	LogNormal(μ=0.82 ,σ=1.45)	Estimated [20]
Sampling frequency	Every 30 days	[27,28]

1.4 Outcome measures

The detection pattern for each simulation falls into one of the following five patterns (Fig 1B).

1. No detection: neither AFP surveillance nor ES detected the poliovirus circulation.
2. AFP surveillance only detected polio patients.
3. AFP surveillance detected the poliovirus circulation earlier than ES.
4. ES detected the poliovirus circulation earlier than AFP surveillance.
5. ES only detected the poliovirus circulation.

Let t_{AFP} and t_{ES} be the timing of the first detection through AFP surveillance and ES, respectively. The lead time of the first detection through ES over APF surveillance (denoted as LT) is defined as $t_{AFP} - t_{ES}$, meaning that a positive value of LT corresponds to the early detection by ES (pattern 4) and a negative value of LT corresponds to the early detection by AFP surveillance (pattern 3). The lead time can only be calculated for patterns 3 and 4.

We used the proportion of each detection pattern among simulations with any poliovirus detection as the primary outcome and excluded no detection pattern to match simulation outcomes with the real-world observations. To inform the quantitative aspect of early detection, we further classified pattern 3 into "< -60 LT" and "-60 ~ -1 LT" categories and pattern 4 into "0 – 59 LT", and "≥60 LT" categories. We refer to the "simulated early detection probability" as the sum of the proportions of "0 – 59 LT", "≥60 LT" and ES only detection patterns.

1.5 Average minimum distance to ES-covered patches and simulated early detection probability.

We quantified the simulated early detection probability varying the number and location of the ES. However, our stochastic simulation took a long time and implementation was complex. We therefore explored a simple alternative measurement to inform the ES site layout strategy. Given importation risk distribution and EIPs in each patch were known, we calculated the weighted average minimum distance to the closest ES-covered patch and quantified the relationship between this measure and the simulated early detection probability.

The average minimum distance to the closest ES-covered patches (d_{ave}) weighted by the importation risks is given as

$$d_{ave} = \sum_{i} r_i \min(\{d_{ij}; j \in S_{ES}\}), \tag{S10}$$

where r_i is the importation risk at patch i for each scenario, d_{ij} is the distance between patch i and patch j, and S_{ES} is the set of patches with the ES.

Since the regional heterogeneity in vaccination coverage can influence the timing of detection, we also consider the average minimum distance weighted by the importation risks and the outbreak probability in each patch ($d_{ave,w}$):

$$d_{ave,w} = \frac{\sum_i r_i P(X \geq 10; R_{e.i}) \min\left(\{d_{ij}; j \in S_{ES}\}\right)}{\sum_i r_i P\left(X \geq 10; R_{e,i}\right)}, \tag{S11}$$

where $P\left(X \geq 10; R_{e,i}\right)$ denotes the outbreak probability of 10 or more infections occurring given the effective reproduction number at patch i in the initial state ($R_{e,i}$, see the definition in the Section 1.3.1). We visualised the relationship between $d_{ave,w}$ and the simulated early detection ability in the main analysis (Fig 5). We arbitrarily chose the value of 10 infections for the cutoff. Assuming the

branching process and the Poisson distribution as the offspring distribution (i.e. no overdispersion), the probability of observing x cases given $R_{e,i}$ follows the Borel-Tanner distribution [37–39].

$$P(X = x; R_{e,i}) = \frac{x^{x-2} R_{e,i}^{x-1} e^{-xR_{e,i}}}{(x-1)!}. \tag{S12}$$

Then, the probability of 10 or more infections occurring is calculated by considering the complement of less than 10 infections occurring.

$$P(X \geq 10; R_{e,i}) = 1 - \sum_{i=1}^{9} P(X = i; R_{e,i}). \tag{S13}$$

The histogram of $R_{e,i}$ in South Africa and the corresponding probability of at least 10 infections happening given a single introduction is shown in Fig S9.

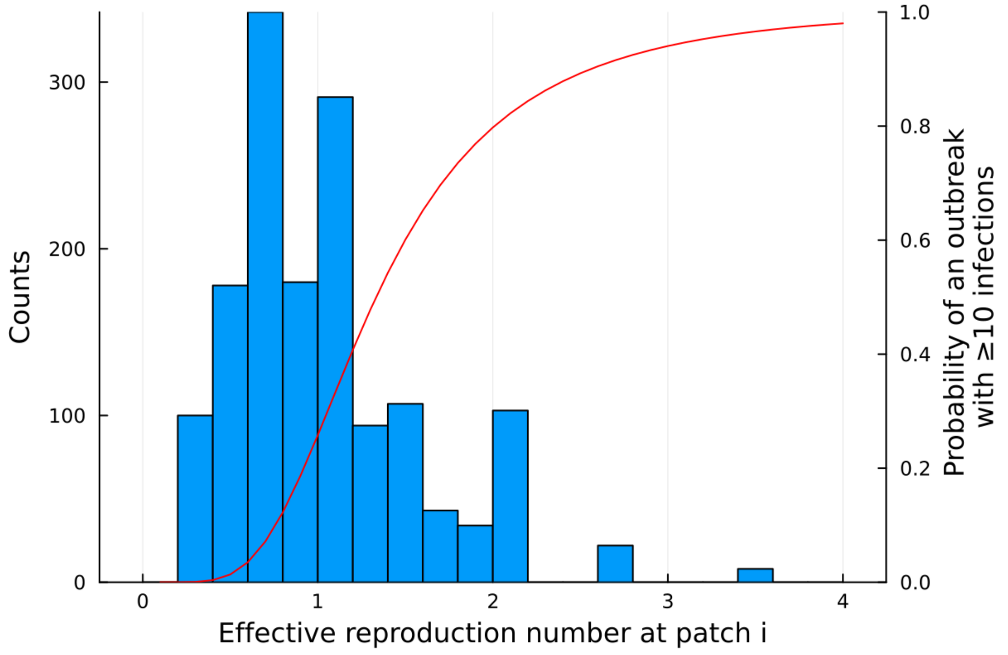

Fig S9. Histogram of the effective reproduction number for each patch and corresponding outbreak probability with ≥10 infections.

2 Supplementary results

2.1 Characteristics for the top 20 populous patches

Table S6. Population size and effective immunisation proportion (EIP) for the top 20 populous patches.

Longitude	Latitude	District of each patch	Population size aged under 5 years old	Cumulative proportion (%)	EIP[d]	Unimmunised population aged under 5 years old
27.77	-26.15	West Rand	88395	3.1	94.9	4508
18.57	-33.82	City of Cape Town	66488	5.5	94.7	3516
30.83	-29.79	eThekwini[a]	55556	7.4	90.3	5408
28.15	-25.96	City of Johannesburg	51209	9.2	93.4	3369
27.96	-26.15	City of Johannesburg	48254	10.9	93.4	3175
30.83	-29.60	iLembe	46696	12.6	75.4	11483
18.57	-34.01	City of Cape Town[b]	40666	14.0	94.7	2151
18.37	-33.82	City of Cape Town	40057	15.4	94.7	2118
27.96	-25.96	City of Johannesburg	37234	16.7	93.4	2450
27.96	-25.39	City of Tshwane	35821	18.0	94.5	1983
28.15	-26.15	Ekurhuleni[c]	35621	19.3	94.1	2110
25.47	-33.82	Nelson Mandela Bay	34878	20.5	91.5	2962
27.77	-26.34	West Rand	27991	21.5	94.9	1427
27.96	-25.77	City of Tshwane	25726	22.4	94.5	1424
28.15	-25.58	City of Tshwane	25023	23.3	94.5	1385
27.77	-26.54	Sedibeng	24300	24.1	91.9	1965
27.77	-25.96	West Rand	20628	24.9	94.9	1052
28.34	-25.58	City of Tshwane	19920	25.6	94.5	1103
28.34	-26.15	Ekurhuleni	19597	26.3	94.1	1161
18.37	-34.01	City of Cape Town	17993	26.9	94.7	952

[a] Closest patch to King Shaka International Airport among the patches in this table. The rank of population size in King Shaka International Airport's patch is 61st.
[b] Cape Town International Airport is in this patch.
[c] Tambo International is in this patch.
[d] Effective immunisation proportion.

2.2 Simulation results under a single patch setting.

We simulated a stochastic SEIR model in a single patch to differentiate between the effects of parameters on the simulated early detection ability of ES attributable to spatial components and those stemming from model behaviours in a single patch. We employed the same model without a meta-population framework using the parameters listed in Table S5. We chose 100,000 population size of children aged under 5 years old and set the population size of all ages as 100,000 divided by the patch-level ES population coverage (p_c).

We visualised the simulated cumulative detection probability over time for the AFP surveillance (dotted lines) and ES (solid lines) in Fig S10. Additionally, we visualised the proportion of any detection of the poliovirus circulation among all simulations including no detection pattern, and the

proportion of each detection pattern given poliovirus circulation is detected (excluding no detection pattern).

Theoretically, ES-related parameters (sampling frequency, ES sensitivity, patch-level ES population coverage) do not influence the simulated cumulative probability of detection for AFP surveillance, which was confirmed by our simulations. Regarding population size, as the population size of children under 5 years old increased, the simulated cumulative probability for AFP surveillance also increased since the absolute value of patients with poliovirus became larger. On the other hand, the simulated cumulative probability for ES decreased because we assumed ES sensitivity was dependent on the incidence rate and a larger denominator resulted in the lower incidence rate.

We re-estimated ES sensitivity parameters with the simulated first detection timing through ES using Equation S5 to validate our assumption that ES sensitivity is proportional to the incidence rate of infectious individuals (Fig S11). We did not include simulated negative environmental samples for the estimation. Through this procedure, we illustrated higher ES sensitivity estimates for a large population size and lower ES sensitivity estimates for a small population size. This model behaviour is consistent with other estimates using the wastewater sample data [40].

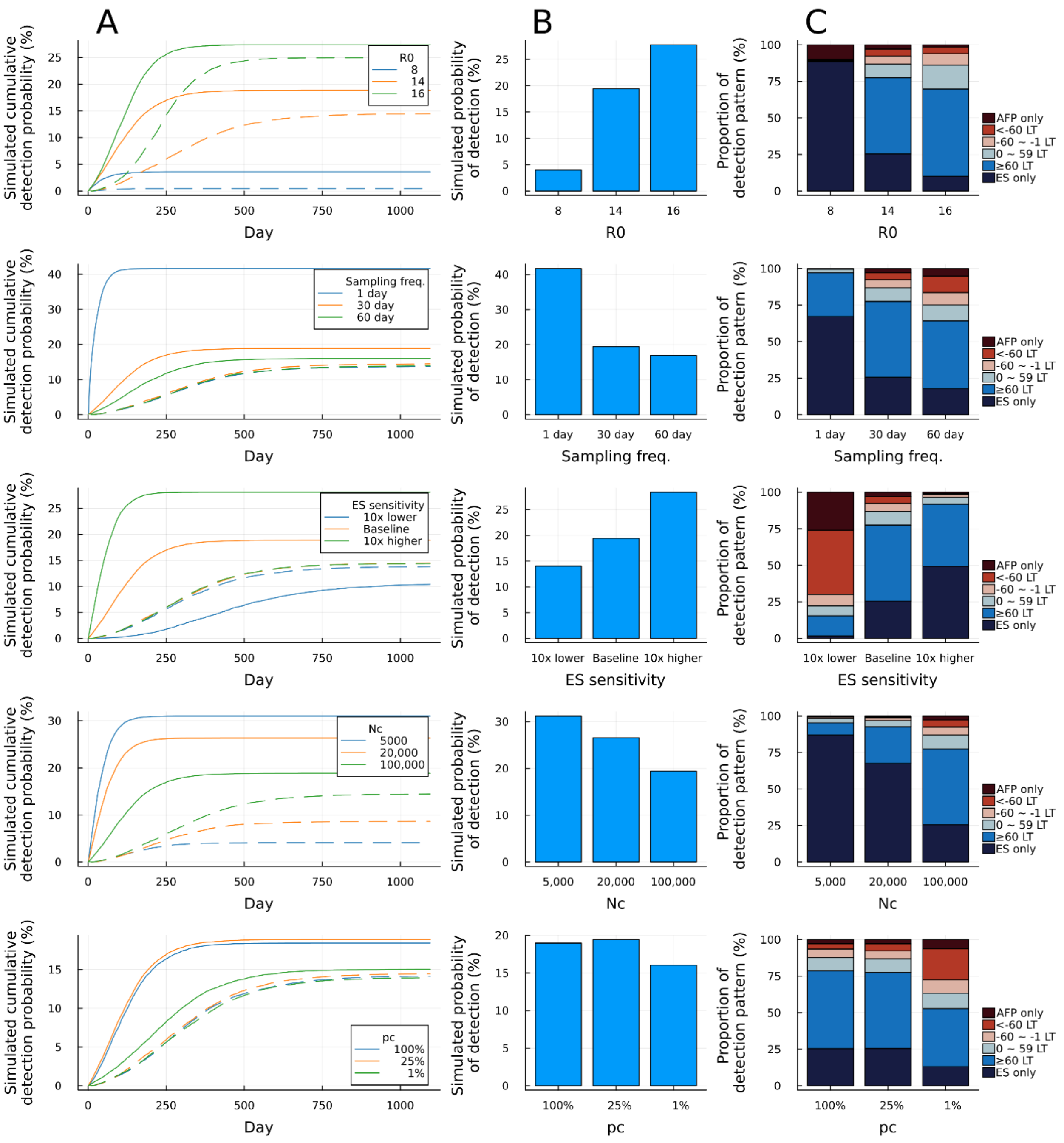

Fig S10. Sensitivity analysis of parameters in a single patch setting. (A) Simulated cumulative detection probabilities. Solid lines and dotted lines represent simulated probability for ES and AFP surveillance, respectively. (B) Simulated probability of detection through either AFP surveillance or ES among all simulations including no detection pattern (%). (C) The proportion of each detection pattern (%) given poliovirus circulation was detected.

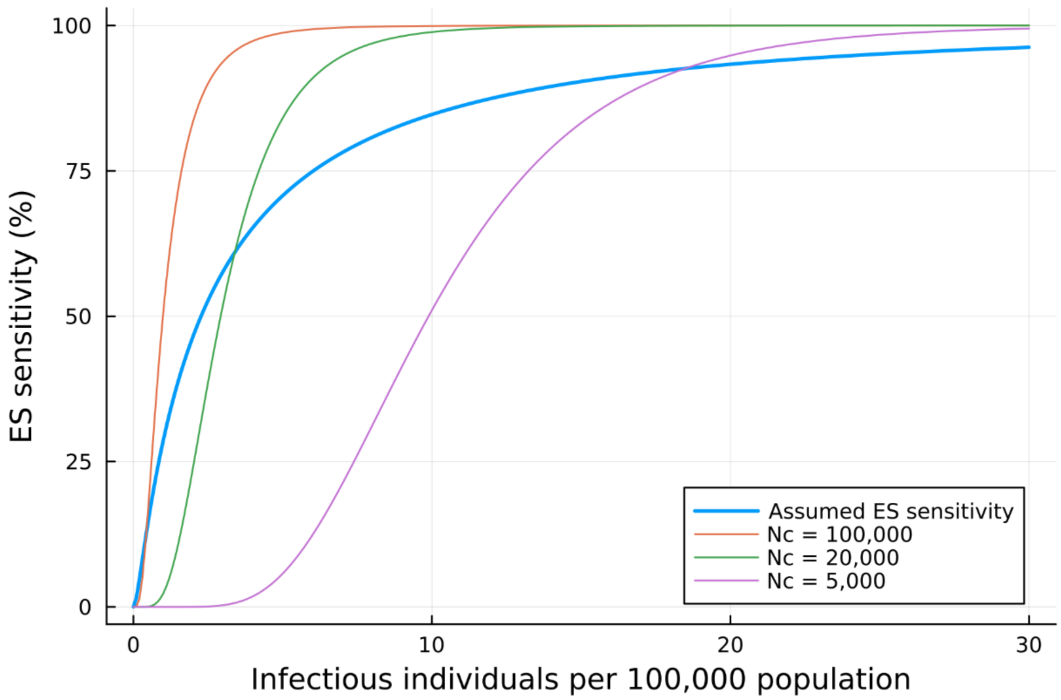

Fig S11. Dose-response curves for estimated ES sensitivity parameters under different population sizes of children under 5 years old (N_c) in a single patch setting.

2.3 Two alternative visualisations for the main analysis

We limited the x-axis for Fig 2 to 160 for the interpretability of the results, and here we visualised the same results where the x-axis represents the national ES population coverage (Fig S12). The national ES population coverage was obtained by the product of the percentage of the population in ES-covered patches and the patch-level ES population coverage (p_c). Therefore, the maximum value of the ES population coverage is matched with our parameter choice for p_c (i.e. 25%). It is noted that the maximum number of ES-covered patches of 1502 corresponds to the national ES population coverage of 25%, and 160 ES-covered patches were close to the plateau of the simulated early detection probability.

We further calculated the proportion of each detection pattern by including the “no detection” pattern (Fig S13) whereas we did not include the “no detection” pattern in the main text figures. Differences in the proportion of the “no detection” pattern would be attributable to the ES layout strategy and heterogeneity in the effective reproduction number in each path (Fig S9).

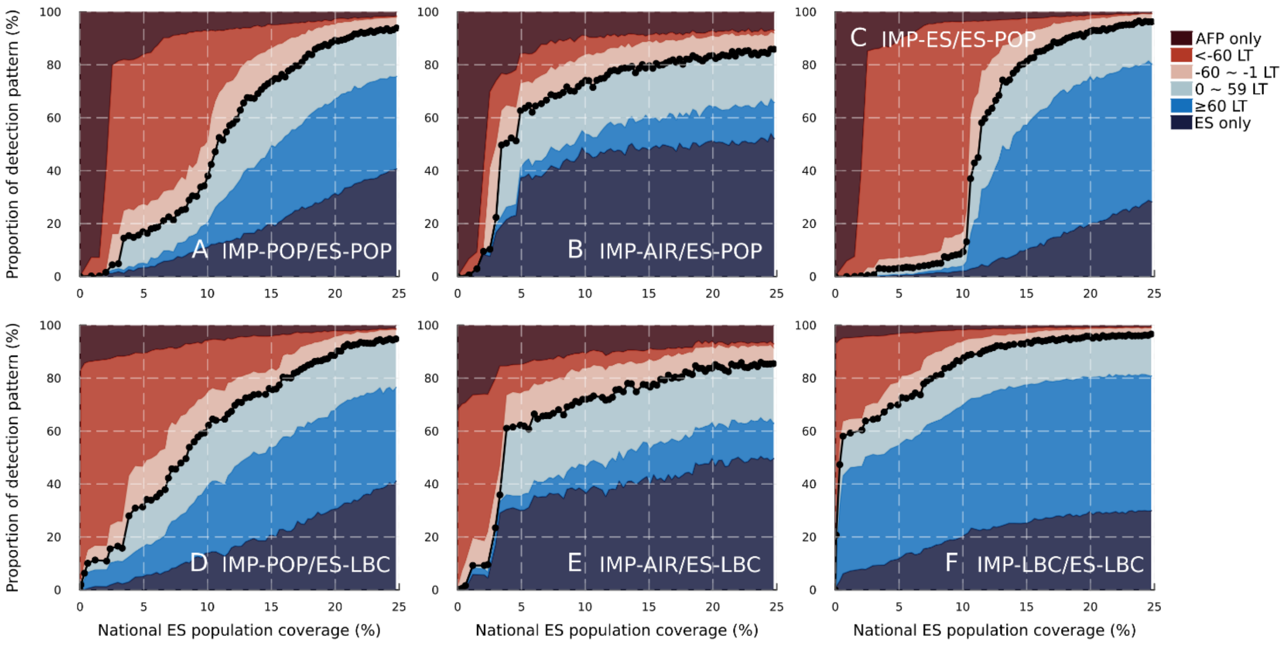

Fig S12. Proportion of each detection pattern (%) against national ES population coverage for six scenarios. The blue-coloured area under the black dotted lines represents the simulated early detection probability, consisting of early detection of ES over AFP surveillance and ES only detection. LT denotes the lead time of poliovirus detection through ES over AFP surveillance.

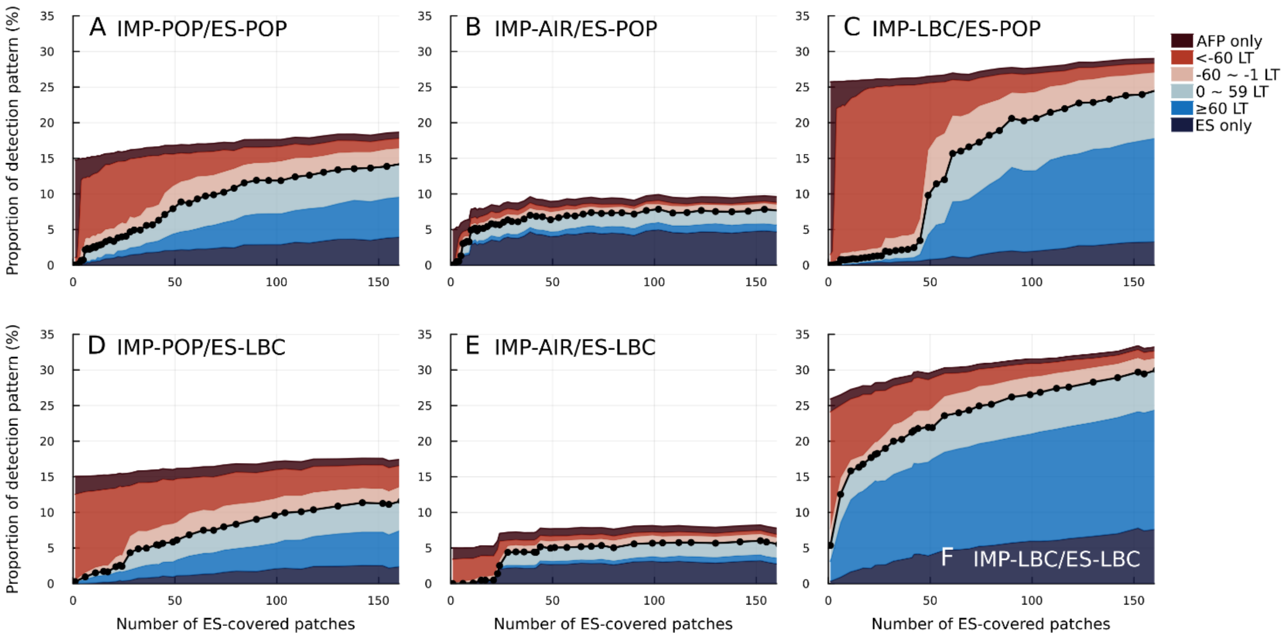

Fig S13. Proportion of each detection pattern (%) including the no detection pattern against the number of ES-covered patches for six scenarios. The non-coloured area represents the simulated probability of detecting poliovirus circulation neither through AFP surveillance nor ES. The blue-colours area under the black dotted lines represents the simulated early detection probability. LT denotes the lead time of poliovirus detection through ES over AFP surveillance.

2.4 Sensitivity analysis on the patch-level ES population coverage for different scenarios

We conducted the sensitivity analysis of the patch-level ES population coverage (p_c) for the other 5 scenarios as well as the main text (Fig 4). We found a similar trend in Fig S14-S15 as in Fig 4. That is, the simulated early detection probability was robust against the number of ES-covered patches but had a large variation against the national ES population coverage.

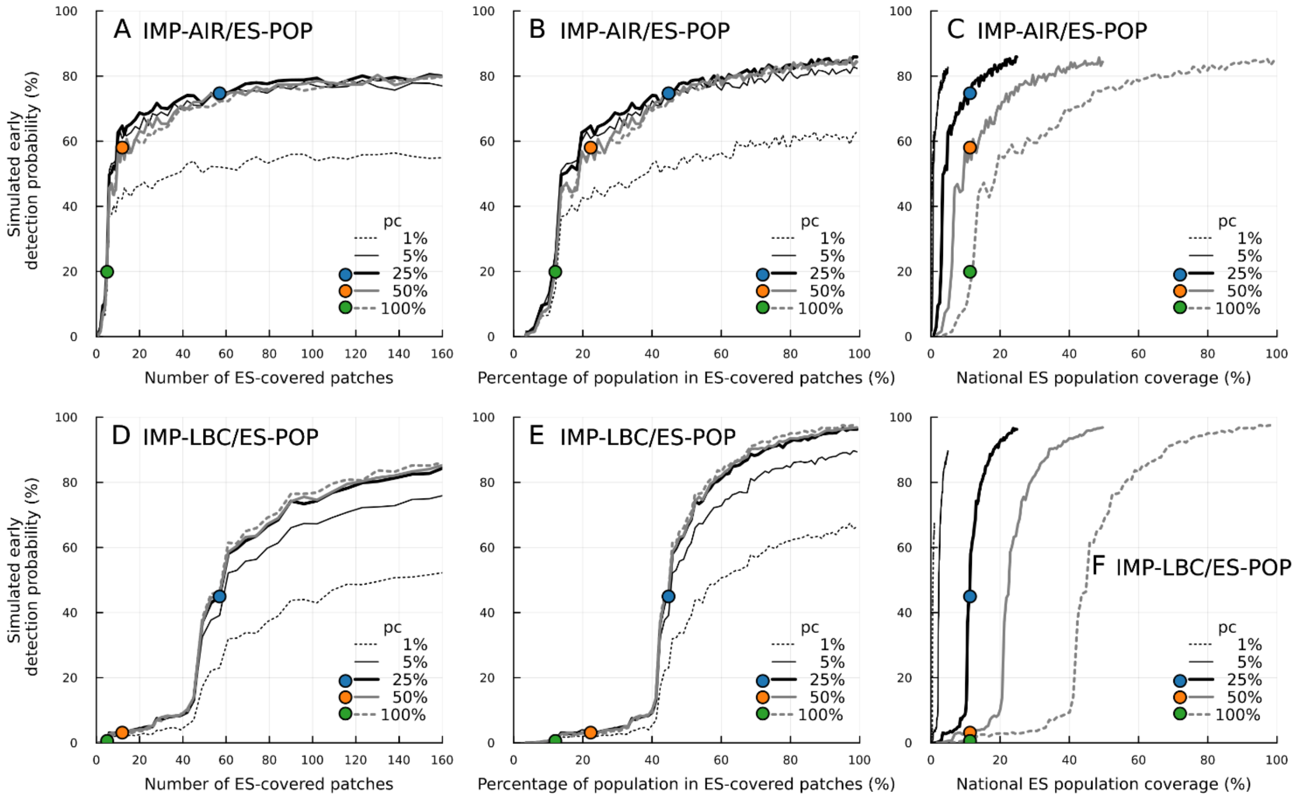

Fig S14. Sensitivity analysis of the patch-level ES population coverage, p_c, for the ES-pop scenarios. (A, B, C) For the IMP-AIR/ES-POP scenario. (C, D, F) For the IMP-LBC/ES-POP scenario. Simulated early detection probability is plotted (A, D) against the number of ES-covered patches, (B, E) against the percentage of the population in ES-covered patches, and (C, F) against the national ES population coverage. The data points represent simulations where the national ES population coverage of the simulated ES layout aligns with the current coverage in South Africa (11.3%), under p_c of 25% (blue), 50% (orange) and 100% (orange). The national ES population coverage is given by the product of p_c and the percentage of the population in ES-coverage patches. It is noted that the maximum number of ES-covered patches is 1502 and the x-axis for (A, D) is limited to a maximum value of 160.

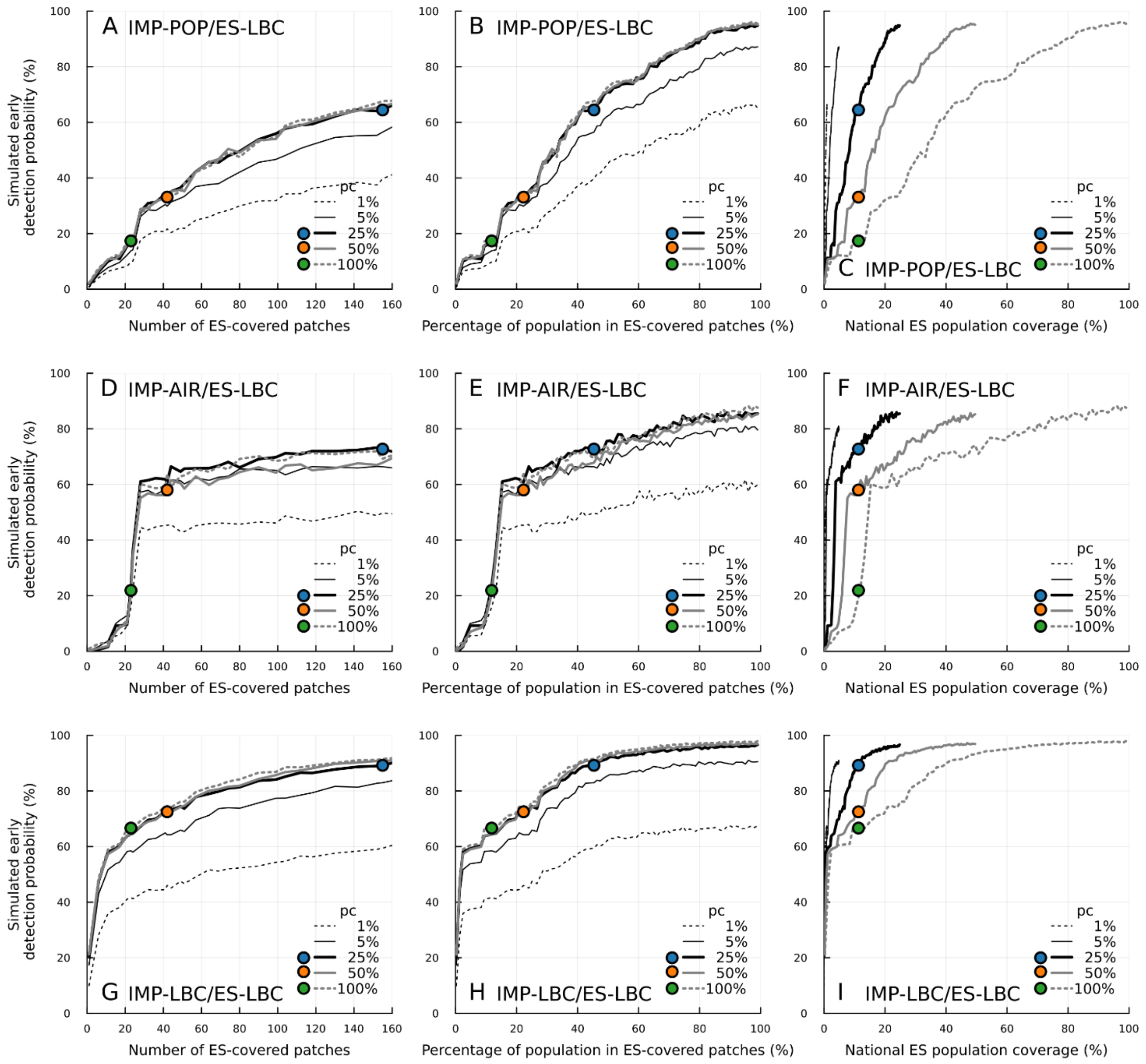

Fig S15. Sensitivity analysis of the patch-level ES population coverage, p_c, for the ES-LBC scenarios. (A, B, C) For the IMP-POP/ES-LBC scenario. (D, E, F) For the IMP-AIR/ES-LBC scenario. (G, H, I) For the IMP-LBC/ES-LBC scenario. Simulated early detection probability is plotted (A, D, G) against the number of ES-covered patches, (B, E, H) against the percentage of the population in ES-covered patches, and (C, F, I) against the national ES population coverage. The data points represent simulations where the national ES population coverage of the simulated ES layout aligns with the current coverage in South Africa (11.3%), under p_c of 25% (blue), 50% (orange) and 100% (orange). The national ES population coverage is given by the product of p_c and the percentage of the population in ES-coverage patches. It is noted that the maximum number of ES-covered patches is 1502 and the x-axis for (A, D, G) is limited to a maximum value of 160.

3 References

1. Tatem AJ. WorldPop, open data for spatial demography. Sci Data. 2017 Jan 31;4(1):170004.

2. Runfola D, Anderson A, Baier H, Crittenden M, Dowker E, Fuhrig S, et al. geoBoundaries: A global database of political administrative boundaries. Tang W, editor. PLoS ONE. 2020 Apr 24;15(4):e0231866.

3. Republic of South Africa Expanded Programme on Immunisation (EIP) national coverage survey report 2020 [Internet]. Department of Health, Pretoria, South Africa; Available from: https://www.health.gov.za/wp-content/uploads/2022/03/National-EPI-Coverage-Survey_Final-full-report-Dec-2020.pdf

4. Chezzi C, Blackburn NK, Schoub BD. Molecular characterisation of type 1 polioviruses associated with epidemics in South Africa. J Med Virol. 1997 May;52(1):42–9.

5. Grassly NC. Immunogenicity and effectiveness of routine immunization with 1 or 2 doses of inactivated poliovirus vaccine: systematic review and meta-analysis. J Infect Dis. 2014 Nov 1;210(suppl_1):S439–46.

6. Molodecky NA, Jafari H, Safdar RM, Ahmed JA, Mahamud A, Bandyopadhyay AS, et al. Modelling the spread of serotype-2 vaccine derived-poliovirus outbreak in Pakistan and Afghanistan to inform outbreak control strategies in the context of the COVID-19 pandemic. Vaccine. 2023 Apr;41:A93–104.

7. Thompson KM, Pallansch MA, Duintjer Tebbens RJ, Wassilak SG, Kim JH, Cochi SL. Preeradication vaccine policy options for poliovirus infection and disease control. Risk Anal. 2013 Apr;33(4):516–43.

8. Ranta J, Hovi T, Arjas E. Poliovirus surveillance by examining sewage water specimens: Studies on detection probability using simulation models. Risk Anal. 2001 Dec;21(6):1087–96.

9. Brouwer AF, Eisenberg JNS, Pomeroy CD, Shulman LM, Hindiyeh M, Manor Y, et al. Epidemiology of the silent polio outbreak in Rahat, Israel, based on modeling of environmental surveillance data. Proc Natl Acad Sci USA [Internet]. 2018 Nov 6 [cited 2024 Aug 14];115(45). Available from: https://pnas.org/doi/full/10.1073/pnas.1808798115

10. Wagner BG, Behrend MR, Klein DJ, Upfill-Brown AM, Eckhoff PA, Hu H. Quantifying the impact of expanded age group campaigns for polio eradication. Bauch CT, editor. PLoS ONE. 2014 Dec 1;9(12):e113538.

11. Post WM, DeAngelis DL, Travis CC. Endemic disease in environments with spatially heterogeneous host populations. Math Biosci. 1983 Apr;63(2):289–302.

12. Hagenaars TJ, Donnelly CA, Ferguson NM. Spatial heterogeneity and the persistence of infectious diseases. J Theor Biol. 2004 Aug;229(3):349–59.

13. Molodecky NA, Blake IM, O'Reilly KM, Wadood MZ, Safdar RM, Wesolowski A, et al. Risk factors and short-term projections for serotype-1 poliomyelitis incidence in Pakistan: A spatiotemporal analysis. Viboud C, editor. PLoS Med. 2017 Jun 12;14(6):e1002323.

14. Voorman A, O'Reilly K, Lyons H, Goel AK, Touray K, Okiror S. Real-time prediction model of cVDPV2 outbreaks to aid outbreak response vaccination strategies. Vaccine. 2023 Apr;41:A105–12.

15. Mangal TD, Aylward RB, Shuaib F, Mwanza M, Pate MA, Abanida E, et al. Spatial dynamics and high risk transmission pathways of poliovirus in Nigeria 2001-2013. Codeço CT, editor. PLoS ONE. 2016 Sep 26;11(9):e0163065.

16. Tebbens RJD, Pallansch MA, Chumakov KM, Halsey NA, Hovi T, Minor PD, et al. Review and assessment of poliovirus immunity and transmission: Synthesis of knowledge gaps and identification of research needs. Risk Anal. 2013 Apr;33(4):606–46.

17. Grassly NC, Fraser C, Wenger J, Deshpande JM, Sutter RW, Heymann DL, et al. New strategies for the elimination of polio from India. Science. 2006 Nov 17;314(5802):1150–3.

18. Casey AE. The incubation period in epidemic poliomyelitis. JAMA. 1942 Nov 14;120(11):805.

19. Liu AB, Lee D, Jalihal AP, Hanage WP, Springer M. Quantitatively assessing early detection strategies for mitigating COVID-19 and future pandemics. Nat Commun. 2023 Dec 20;14(1):8479.

20. Wu F, Xiao A, Zhang J, Moniz K, Endo N, Armas F, et al. Wastewater surveillance of SARS-CoV-2 across 40 U.S. states from February to June 2020. Water Research. 2021 Sep;202:117400.

21. McCarthy KA, Chabot-Couture G, Shuaib F. A spatial model of Wild Poliovirus Type 1 in Kano State, Nigeria: calibration and assessment of elimination probability. BMC Infect Dis. 2016 Sep 29;16(1):521.

22. Fine PEM, Carneiro IAM. Transmissibility and persistence of oral polio vaccine viruses: Implications for the Global Poliomyelitis Eradication Initiative. Ame J Infect. 1999 Nov 15;150(10):1001–21.

23. Kalkowska DA, Franka R, Higgins J, Kovacs SD, Forbi JC, Wassilak SGF, et al. Modeling Poliovirus Transmission in Borno and Yobe, Northeast Nigeria. Risk Anal. 2021 Feb;41(2):289–302.

24. Kalkowska DA, Duintjer Tebbens RJ, Thompson KM. Modeling Strategies to Increase Population Immunity and Prevent Poliovirus Transmission in the High-Risk Area of Northwest Nigeria. The Journal of Infectious Diseases. 2014 Nov 1;210(suppl_1):S412–23.

25. Howard W, Moonsamy S, Seakamela L, Jallow S, Modiko F, du Plessis H, et al. Sensitivity of the acute flaccid paralysis surveillance system for poliovirus in South Africa, 2016–2019. J Med Microbiol. 2021 Oct 21;70(10):001441.

26. Gerloff N, Sun H, Mandelbaum M, Maher C, Nix WA, Zaidi S, et al. Diagnostic Assay Development for Poliovirus Eradication. J Clin Microbiol. 2018 Feb;56(2):e01624-17.

27. Hamisu AW, Blake IM, Sume G, Braka F, Jimoh A, Dahiru H, et al. Characterizing environmental surveillance sites in Nigeria and their sensitivity to detect poliovirus and other enteroviruses. J Infect Dis. 2022 Apr 19;225(8):1377–86.

28. Global polio eradication initiative. Guidelines on environmental surveillance for detection of polioviruses [Internet]. 2023. Available from: https://polioeradication.org/wp-content/uploads/2016/07/GPLN_GuidelinesES_April2015.pdf

29. Duintjer Tebbens RJ, Pallansch MA, Cochi SL, Ehrhardt DT, Farag NH, Hadler SC, et al. Modeling poliovirus transmission in Pakistan and Afghanistan to inform vaccination strategies in undervaccinated subpopulations. Risk Anal. 2018 Aug;38(8):1701–17.

30. Thompson KM, Pallansch MA, Duintjer Tebbens RJ, Wassilak SG, Kim JH, Cochi SL. Preeradication vaccine policy options for poliovirus infection and disease control. Risk Anal. 2013 Apr;33(4):516–43.

31. Grassly NC. Immunogenicity and effectiveness of routine immunization with 1 or 2 doses of inactivated poliovirus vaccine: Systematic review and meta-analysis. J Infect Dis. 2014 Nov 1;210(Suppl 1):S439–46.

32. Debré R, Duncan D, Enders JF, Freyche MJ, Gard S, Gear J, et al. Poliomyelitis [Internet]. World Health Organization; 1955 [cited 2023 Aug 6]. Available from: https://apps.who.int/iris/handle/10665/41659

33. Sallah K, Giorgi R, Bengtsson L, Lu X, Wetter E, Adrien P, et al. Mathematical models for predicting human mobility in the context of infectious disease spread: introducing the impedance model. International Journal of Health Geographics. 2017 Nov 22;16(1):42.

34. Nathanson N, Kew OM. From emergence to eradication: The epidemiology of poliomyelitis deconstructed. Am J Epidemiol. 2010 Dec 1;172(11):1213–29.

35. Tesfaye B, Sowe A, Kisangau N, Ogange J, Ntoburi S, Nekar I, et al. An epidemiological analysis of acute flaccid paralysis (AFP) surveillance in Kenya, 2016 to 2018. BMC Infect Dis. 2020 Aug 18;20(1):611.

36. O'Reilly KM, Grassly NC, Allen DJ, Bannister-Tyrrell M, Cameron A, Carrion Martin AI, et al. Surveillance optimisation to detect poliovirus in the pre-eradication era: a modelling study of England and Wales. Epidemiol Infect. 2020;148:e157.

37. Farrington CP. Branching process models for surveillance of infectious diseases controlled by mass vaccination. Biostatistics. 2003 Apr 1;4(2):279–95.

38. Nishiura H, Yan P, Sleeman CK, Mode CJ. Estimating the transmission potential of supercritical processes based on the final size distribution of minor outbreaks. J Theor Biol. 2012 Feb;294:48–55.

39. Blumberg S, Funk S, Pulliam JRC. Detecting differential transmissibilities that affect the size of self-limited outbreaks. Wilke CO, editor. PLoS Pathog. 2014 Oct 30;10(10):e1004452.

40. Li Q, Lee BE, Gao T, Qiu Y, Ellehoj E, Yu J, et al. Number of COVID-19 cases required in a population to detect SARS-CoV-2 RNA in wastewater in the province of Alberta, Canada: Sensitivity assessment. J Environ Sci. 2023 Mar;125:843–50.
