# Supplementary figures and images for: Assessing early detection ability through spatial arrangements in environmental surveillance for poliovirus: A simulation-based study
Source: PLoS One. 2025 Jul 9;20(7):e0325789. doi: 10.1371/journal.pone.0325789 (PMC12240349; doi:10.1371/journal.pone.0325789)

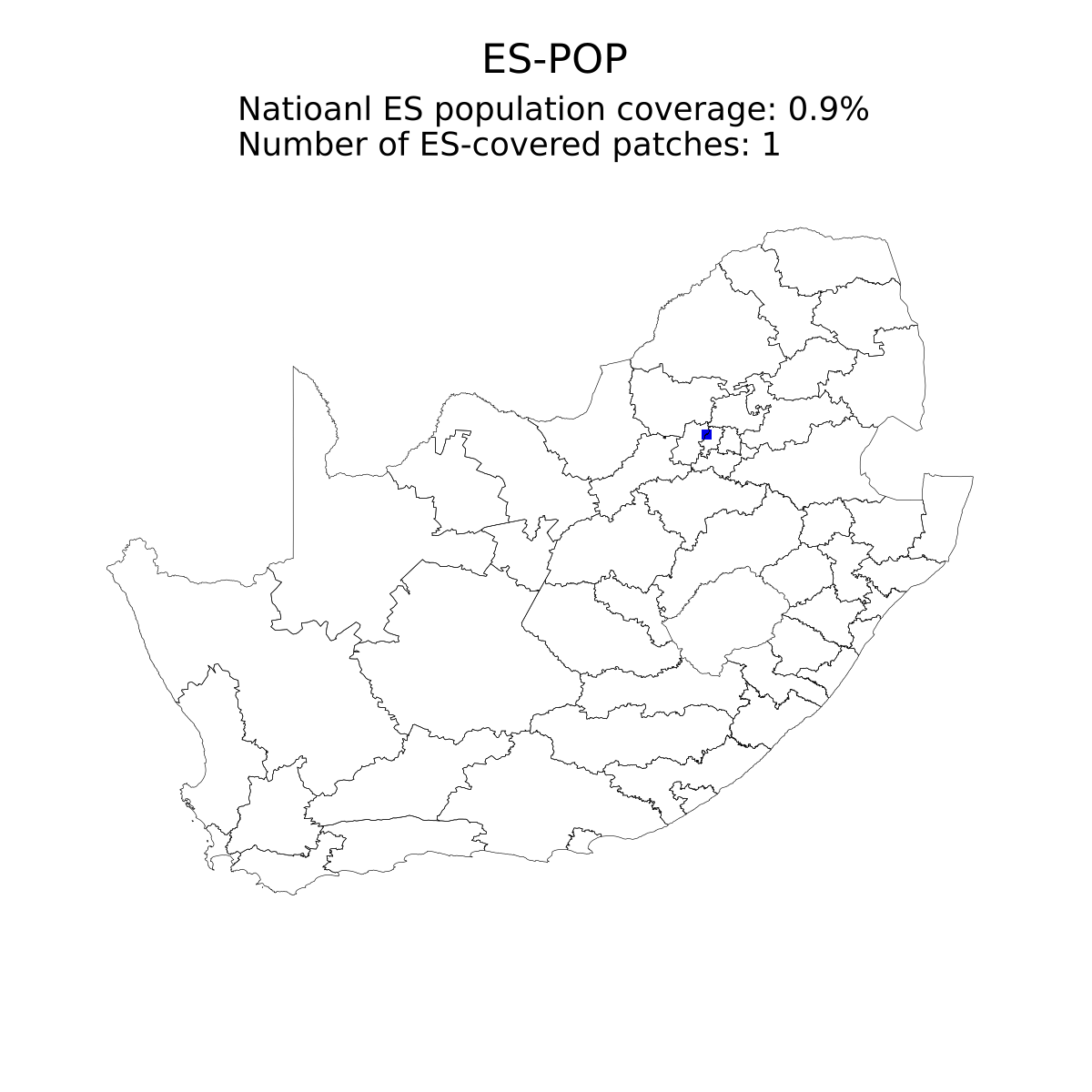

Supplement: S1 Video — ES sites were implemented in descending order of the population size of each patch. Blue squared areas represent patches covered by ES sites. (GIF) [file pone.0325789.s002.gif]

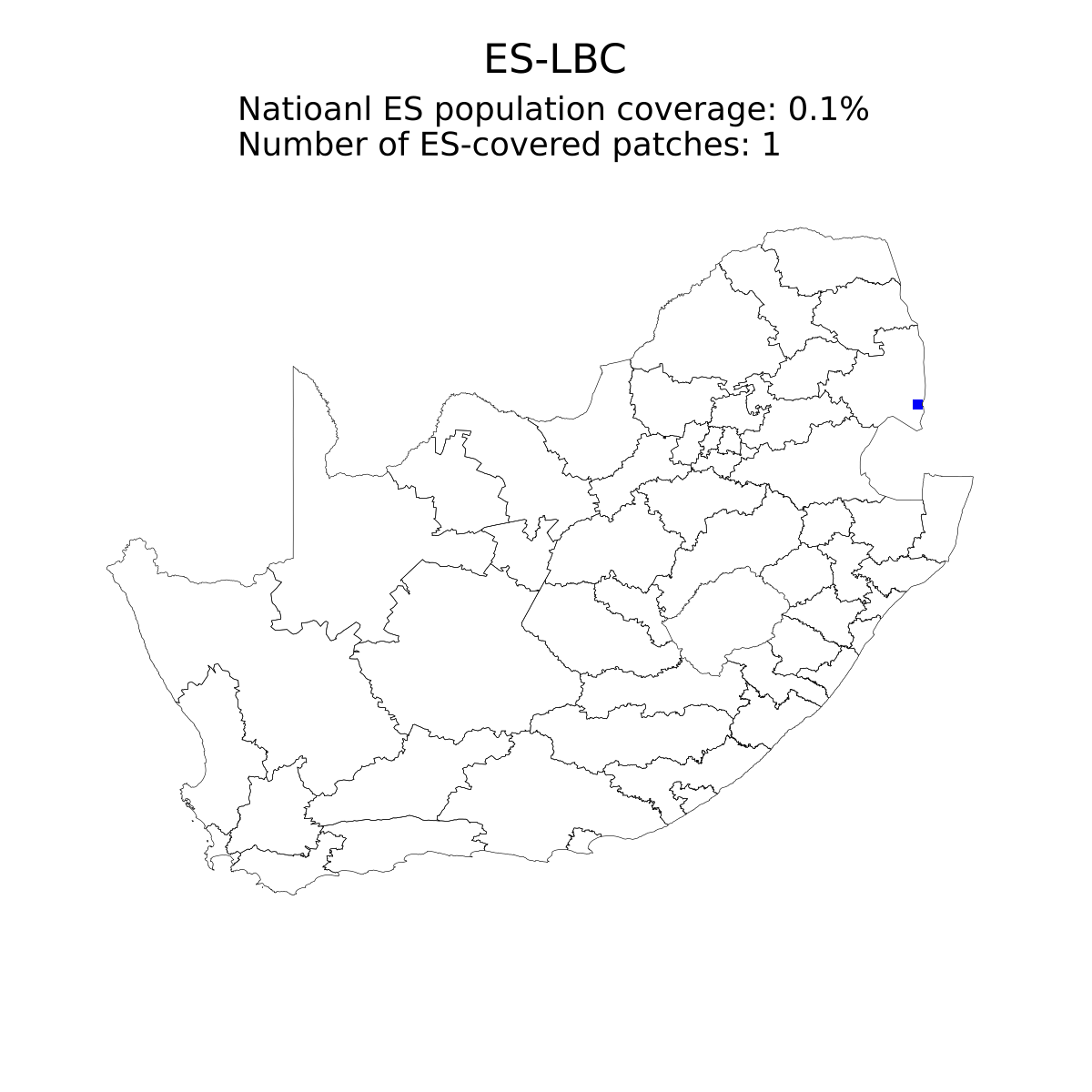

Supplement: S2 Video — ES sites were first implemented in a patch with a high importation risk via land border crossing from Mozambique. Blue squared areas represent patches covered by ES sites. (GIF) [file pone.0325789.s003.gif]
