# Supplementary material for: Assessing early detection ability through spatial arrangements in environmental surveillance for poliovirus: A simulation-based study
Source: PLoS One. 2025 Jul 9;20(7):e0325789. doi: 10.1371/journal.pone.0325789 (PMC12240349; doi:10.1371/journal.pone.0325789)
Supplement: Attachment — Submitted filename: Response to Reviewers.pdf [file pone.0325789.s004.pdf]

Point-by-point responses

Reviewer #1: Summary: This interesting study used simulation modeling to highlight and explore the potential of early detection of polio outbreaks by environmental surveillance (ES). The authors demonstrated how the geography of importation affects detection probability and the utility of ES as an early detection tool. The introduction is strong, and the conclusions are sensible and useful. I have some suggestions that may improve the clarity of the methods and results for the reader. I think the paper will be a useful addition to the ES literature.

Comments

1. I think that this paper suffers from trying to report the results before the methods, because the authors end up having to say the methods twice, and incompletely/confusingly the first time. I recommend reordering the paper with the methods first, so that the results can be more easily understood.

Response 1: We thank the reviewer for pointing out the current insufficient explanation of the Methods and for suggesting reordering the paper. Following the suggestion, we have reordered the Methods first, restructured the subsections to avoid duplicated descriptions, and revised the entire sentences in the Method and Results to ensure clarity.

Action: After reordering the Methods first, we have changed the following major points. Please refer to the changes to the Method in the attached manuscript where revised sentences were coloured in red.

- 1) We revised the last paragraph of the Introduction to ensure natural sentence flow to the Methods.
- 2) We moved the details of the metrics or equations to the supplementary material and emphasised more general explanations and assumptions for those methods in the main manuscript. Specifically, we moved the following contents to the supplementary: the calculation of effectively immunised proportions (EIPs), and Equations (2) and (3) in the previous version.
- 3) We have included more details of simulations and outcomes in 'Model implementation and outcomes'. Also, we have included the motivation of each importation risk distribution and ES site layout strategy in the 'Scenarios' subsection.

Because of reordering our manuscripts, we have deleted the following sentences from the Results to avoid duplicated descriptions in the Methods.

(after page 14, lines 205) "*To investigate the impact of the exclusion of the 'no detection pattern' on the proportion of detection patterns, we visualised those proportions including the 'no detection' pattern in Fig S13 in S1 text. We found significant differences in the simulated probability of at least one detection of poliovirus either through ES or AFP surveillance (coloured areas), which would be attributable to heterogeneity in the effective immunisation proportions.*"

(after page 18, lines 394) "*since our stochastic simulations required a long computation time. We calculated the average minimum distance from patches with importation to ES-covered patches weighted by outbreak probability. The outbreak probability in the present study is defined as the probability of 10 or more infections occurring given a single introduction, considering the effective immunisation proportion (EIP) in each patch (Fig S9 in S1 text). This metric can be calculated if importation risks and ES site layout are available. Intuitively, early detection probability is likely to be higher if ES-covered patches are placed near locations for importation. In this exercise, we wanted to explore whether assumptions on importation and ES placement consistently influenced this property.*"

2. Figure 1. I had trouble processing the image associated with IMP-LBC. First, it wasn't immediately clear visually that you were showing South Africa and Mozambique. Second, I think that the intention is to

highlight transmission risk along the South Africa-Mozambique border, but it seems that only one of the three arrows is across the border. Perhaps you could show South Africa the same size as in the rest of the bottom row of figures, with the border of Mozambique outlined and with arrows crossing the border?

Response 2: We thank the reviewer for suggesting the figure improvement. We kept the same map size for South Africa in IMP-LBC as other importation risk distributions and made the arrows cross the borders. In addition, we modified the caption of Fig 1 to make the figure more understandable.

Action: We have revised Fig 1 and its caption as follows (page 9, lines 202-224).

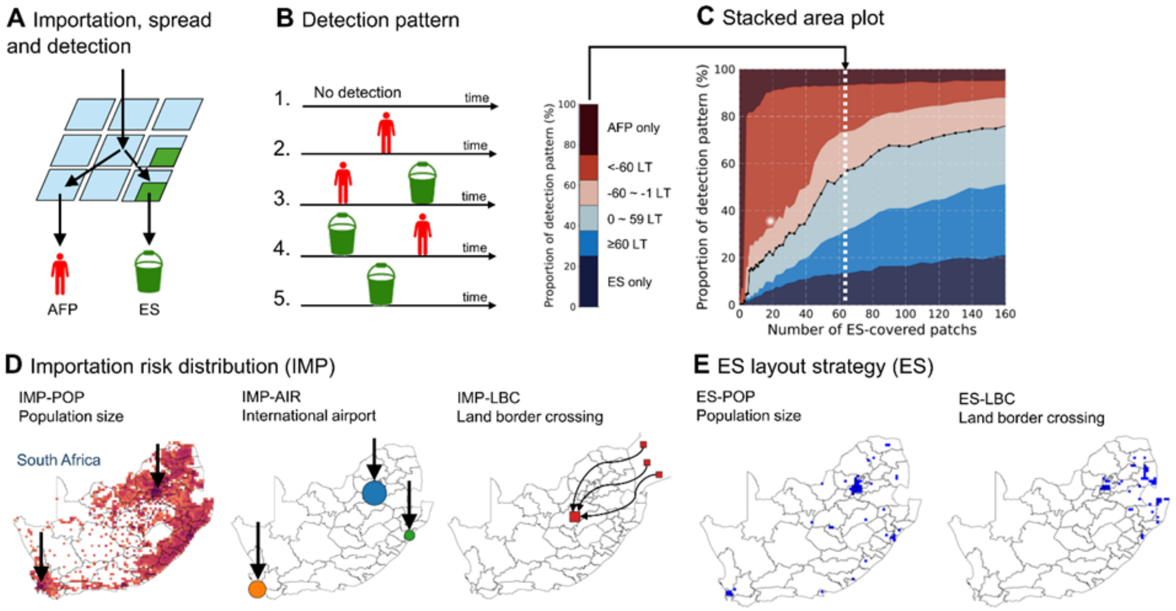

*"**Fig 1. Schematic illustration of study settings.** (A) Simulated importation of wild poliovirus serotype 1, spread described by the meta-population model, and detection through acute flaccid surveillance (AFP) operated in all patches and environmental surveillance (ES) operated in selected patches (in green). (B) Five detection patterns: i) No detection, ii) AFP only detection, iii) earlier detection through AFP surveillance than ES, iv) earlier detection through ES than AFP surveillance v) ES only detection. Detection patterns of iii) and iv) are further classified based on the lead time (LT) of the first detection through ES over AFP surveillance. (C) Stacked area plot of the proportion of each detection pattern against the number of ES-covered patches. (D) Illustration for the generating process of importation risk distributions: i) 'Population size'-based importation risk distribution (IMP-POP). Squares in orange-red colouration represent the population size of each patch in South Africa, which is assumed proportional to importation risks; ii) 'International airport'-based importation risk distribution (IMP-AIR). Each circle denotes the international airport in South Africa with a size proportional to the international inbound travel volume, which is associated with importation risks: Blue, O.R. Tambo International Airport; Orange, Cape Town International Airport; Green, King Shaka International Airport; iii) 'Land border crossing'-based importation risk distribution (IMP-LBC). Arrows illustrate population movement from multiple source patches in Mozambique to a single destination patch in South Africa, sum of which is assumed proportional to importation risk for the destination patch in South Africa. (E) ES site layouts when the simulated national ES population coverage matched the observed one under i) 'Population size'-based ES site layout strategy (ES-POP) and ii) 'Land border crossing importation risk'-based ES site layout strategy (ES-LBC). Blue squares represent ES-covered patches. National and district borders for the maps were drawn with geoBoundaries under the CC-BY 4.0 license [27]."*

3. Figure 1 C and Figure 2. In Figure 1B you describe “no detection” as an option, but it’s not included in these results figures. Was “no detection” not an outcome that was observed? Ah, later you say that it was an outcome but that it’s included in the supplementary material. It’s possible that I missed it, but, as a reader, I could have used some up front notification and justification of why the “no detection” was not being included. Was it because the importation scenario is non-modifiable? Yes, the more that I think about it, the more that I think it would be helpful for you to first talk about (or show) the “no detection” vs “any detection” by scenario before getting into the lead-time results.

Response 3: We apologise for the confusion and thank the reviewer for suggesting including an additional explanation of it. In the revised manuscript, we first explained the explanations of simulation outcomes (e.g. ‘No detection’ or AFP only detection) with simulation implementation and then explained the scenarios, followed by the Result. We removed ‘No detection’ from our main results because it is difficult/impossible to observe ‘No detection’ linked to each importation in the real world and we would like to present results more aligned with the real-world observation.

Action: We have included a description of why we excluded ‘No detection’ pattern from the denominator when calculating the proportions of each detection pattern (page 8, lines 193-197).

“We calculated a proportion of each category based on 10,000 simulations after we excluded “No detection” category as a primary outcome since we were unable to confirm a failure of poliovirus detection for each importation in the real world (see Fig S13 in S1 text for results including “No detection” category).”

4. Figure 2. The scenario abbreviations are cumbersome. Perhaps writing titles for the subpanels would be clearer.

Response 4: We appreciate the reviewer for the comment. After careful consideration, we retained the usage of abbreviations as it is since spelling out those abbreviations for both assumptions (i.e. “importation risk distributions” and “ES site layout strategies”) requires much space, which would make it harder for readers to read the figure eventually. Also, we considered removing the abbreviations from the caption and steering readers to refer to the “Scenarios” subsection in the Methods. However, doing so also might cause difficulty in understanding the abbreviations. For example, “LBC” would be hard to grasp the meaning at first glance without explaining ‘land border crossing’. In the end, we have decided to keep the figure and its caption as it is.

To ensure clarity, we prepared the ‘Scenarios’ subsection specifically, and Fig 1 caption was revised to show the generating process of each risk distribution and the resulting ES site layout from each ES site layout strategy (See Response 2).

5. Interestingly, compared to the IMP-POP scenarios, there was a greater proportion of both AFP only and ES only detection in the scenarios. Can you say more about why that’s happening? (Probably fewer cases means harder to detect through any means?) It comes up again with the “low R0” sensitivity analysis, so it’s worth orienting the reader to.

Response 5: We thank the reviewer for raising the question. Since we did not fully understand which scenario you were comparing to the IMP-POP scenario, we responded to the question assuming the reviewer was comparing the IMP-AIR to the IMP-POP.

In general, an outbreak with a small final size resulted in the AFP only or ES only detection patterns since AFP surveillance detects a case only with a probability of 1/200 and limited geographical spread due to a

small size of outbreaks are likely not to be caught by ES. This tendency can be confirmed through the sensitivity analysis of R0 in a single patch simulation (Fig S10C, top). Then, the high proportion of AFP only or ES only detections for IMP-AIR indicates that patches with high importation risks in IMP-AIR are likely to have higher vaccination coverage compared to other scenarios. This can also be confirmed by the high proportion of "No detection" in IMP-AIR in Fig S13.

Action: We have added the paragraph after Fig 2 to explain the reason for the high proportion of AFP only and ES only detection patterns (pages 13-14, lines 304-309).

"*A high proportion of AFP only or ES only detection patterns in IMP-AIR indicate that patches with high importation risks in IMP-AIR are likely to have a high vaccination coverage, resulting in a small outbreak. This can be confirmed through the sensitivity analysis of R0 within a single patch (Fig S10 in S1 text), showing low R0 leads to high AFP only or ES only detections, and the results including "No detection" category (Fig S12 in S1 text), showing the highest proportion of "No detection" in IMP-AIR.* "

6. The R0 values (10-18) seem high to me. I normally think of polio as being in the 5-7 range, though I'm not an expert on polio specifically. Additionally, you haven't described in the Methods how R0 is connected to the parameterization of the model (is changing R0 just changing beta?), nor have you described/justified the spread of the values of the parameters in the sensitivity analyses performed.

Response 6: We thank the reviewer for questioning R0 assumptions. As the reviewer pointed out, our assumption of R0 is higher than other modelling studies on poliovirus. For example, the papers modelling polio dynamics in Nigeria assumed R0 of 7.5-8.0 for WPV1,[1,2] and another study fitting to the incidence of polio in northern Nigeria yielded a R0 value of 5-10 (or higher) depending on vaccine coverage.[3] We chose R0 of 14 for a baseline since we adjusted the average effective reproduction number (average Re,i, calculated by R0 x effective immunisation proportion at i weighted by population size at patch i) to be slightly above one, and then the distribution of Re,i in each patch (Fig S9) is not unrealistic considering that the effective reproduction number estimates in India from 1997-2006 sometimes reached at three.[4] Since other modelling studies used R0 of 8.0 for their study, we have changed the range of R0 from 10-18 to 8-16 to cover that value. If we chose R0=8, 14, and 16, the average Re,i in our South Africa setting becomes 0.64, 1.13, and 1.29, respectively, and at least R0=14 was observed (although an upper limit) in the previous literature.[5]

Regarding the second point, we related R0 and β by simply using the equation R0= β/γ (where 1/γ represents an infectious period), and just changing β linearly in simulations.

We considered we should clarify the rationale for these assumptions in the main manuscript so that we have revised the method description as follows.

Action: We have added the paragraph describing R0 assumptions and the relationship between R0 and β (page 7, lines 151-157).

"*We calculated β from the relationship R0=β/γ where R0 corresponds to the basic reproduction number and 1/γ corresponds to a latent period. We also define the effective reproduction number in the initial state at patch i (Re,i) as R0 multiplied by EIP at patch i, representing the reproduction number for imported cases. We assumed a relatively higher R0 of 14 compared to previous estimates [32,33] and this assumption resulted in 1.13 of the average Re,i across patches, and most Re,i centred around one with a maximum around three (Fig S9 in S1 text). Additional details can be found in S1 text and parameters used in our model were summarised in Table S5 in S1 text.*"

We have also clarified the corresponding average Re,i for each R0 in the sensitivity analysis (page 11, lines 263-264).

"We performed the sensitivity analysis of R0 (ranging from 8 to 16, corresponding to the average Re,i of 0.80 to 1.45), travelling rate between patches (α), sampling frequency and ES sensitivity for the IMP-POP/ES-POP scenario."

We have updated the Fig 3 to include R0 of 8 in the sensitivity analysis.

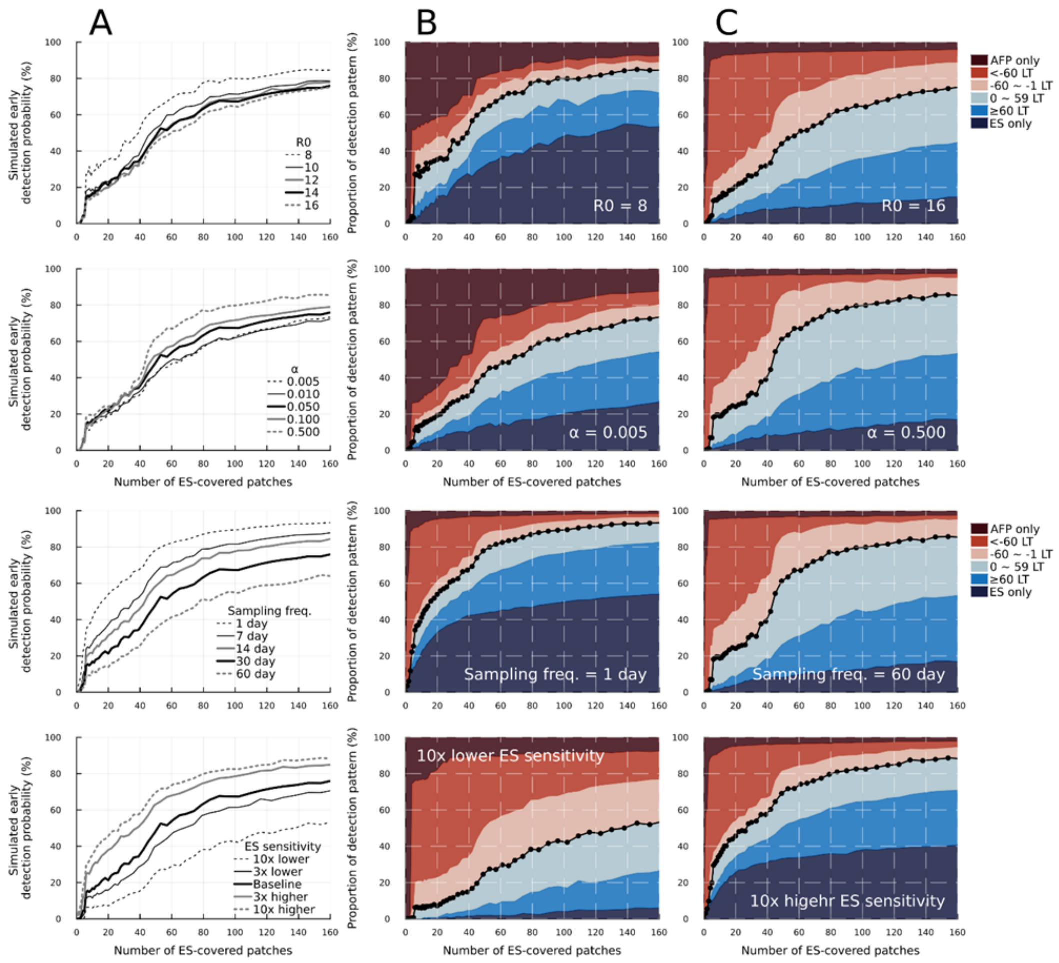

***"Fig 3. Sensitivity analysis of the basic reproduction number (R0), travelling rate between patches (α), sampling frequency and ES sensitivity for the IMP-POP/ES-POP scenario.** (A) Simulated early detection probability for different parameters against the number of ES-covered patches. Thick black lines represent results with the same parameter value as in the main analysis. (B, C) Stacked area plot for the smallest and largest sensitivity parameter values. LT corresponds to the lead time of the first poliovirus detection through ES over AFP surveillance and 'sampling freq.' corresponds to the sampling frequency."*

7. The values of alpha in the Results are fairly meaningless to the reader. What does it mean, practically, to go from alpha= 0.05 to 0.5?

Response 7: We thank the reviewer for providing an opportunity to elaborate on this point. In our model formulation, *α* represents a proportion of individuals outgoing from one patch and can be expressed as Ti/Ni where Ti represents the total number of individuals outgoing from patch i and Ni represents the total

number of population at patch i. Practically, increasing *α* from 0.05 to 0.5, meaning that we assume 50% of the population are always moving out from their residence place because of commuting to work or school. We assumed 0.05 for the main analysis based on the literature fitting the radiation model to cholera incidence (i.e. disease with faecal-oral transmission route) in Haiti by Sallah et al.,[6] and we varied parameters to include values of *α* presented in that study. We have clarified the meaning of *α* and modified the interpretation of the sensitivity analysis result.

Action: We have clarified the meaning of *α* in the method section (page 6, lines 143-144).
"*α corresponds to the travelling rate, representing a proportion of individuals outgoing from one patch, and* n_{ji} *denotes*"

We modified the result description to express that α is less influential on the early detection ability considering a very high mobilisation assumption (page 16, lines 335-337).

"*Even under very high population movement setting by assuming α to be 0.50 (meaning 50% of individuals outgoing from one patch because of, for example, commuting to work or school), we observed only a 15% increase in simulated early detection probability after the number of ES-covered patches exceeded 50.*"

Reviewer #2: The paper "Assessing early detection ability through spatial arrangements in environmental surveillance for poliovirus: a simulation-based study" is a well written and technically sound paper. Below are some comments to the authors:

1. In Fig 1. (D), the IMP-LBC map seems a bit different than the others (I am guessing the authors are showing a subarea of the whole map). Can you please explain which region the map refers to? It will be nice to have a similar map to the rest for easy understanding.

Response 1: We thank the reviewer for the suggestion to improve the figure. We intended to show the mobilisation from Mozambique to South Africa and how this movement is linked to importation risks by showing IMP-LBC in Fig 1D. We have added the country name next to the figure (IMP-POP) and kept using the same country map for IMP-LBC risk distribution to avoid confusion. In addition, we have revised the figure caption for better understanding. Please refer to Reviewer 1 Response (2) for our revision.

2. It seems natural that ES strategy that aligns well with the IMP route will perform better. For instance, the ES-POP strategy covers the IMP-AIR pretty well, as the airports are located in high-density locations, hence the result is supposed to be good. In other words, IMP-Air can be considered as a subset of the IMP-POP. Can you justify the inclusion of this scenario IMP-Air? Also, the authors' contribution beyond this natural alignment is not clear.

Response 2: We thank the reviewer for the comment. As the reviewer pointed out, we also expected the IMP-AIR/ES-POP to perform better due to the well-overlapped importation risks and ES sites. Since visiting or returning travellers from outside South Africa can move around for a long distance during their latent period (assumed to be 4 days), we considered the real-world importation risk via international air passengers to fall somewhere between IMP-POP and IMP-AIR, which led us to test both assumptions.

Regarding the natural alignment, our interest is to quantitatively assess how large-scale ES implementation is required to attain enough early detection ability. For example, the observed national ES coverage in South Africa (11.3%) corresponds to 58 ES-covered patches within our model and with this number of ES-covered patches, we expect 50% of early detection in IMP-POP whereas as high as 88% for IMP-AIR, highlighting the importance of precise importation risk assessment. In addition, our results suggest qualitatively different

relationships between the number of ES sites and expected early detections, which should be taken into account for designing ES in the real world.

3. Lines 182-185, it's hard to interpret whether the result is good or bad. More clarification will be helpful.

Response 3: We thank the reviewer for the comment. We revised the sentence to reflect our intentions more clearly.

Action: We have revised the sentence as follows (pages 12-13, lines 286-292).

"*We can expect a high early detection ability (i.e. more than 50%) from a small number of ES sites (i.e. covering 6 to 8 patches by ES) if importation risks were confined to limited geographical areas and ES was effectively implemented to cover those areas (e.g. IMP-AIR/ES-POP and IMP-LBC/ES-LBC, shown in Fig 2B and 2F). In comparison, if ES-covered areas were not matched with geographical areas with high importation risks (e.g. IMP-LBC/ES-POP and IMP-AIR/ES-LBC, shown in Fig 2C and 2E), we did not expect the implementation of ES in 10 to 20 patches to detect poliovirus circulation earlier than AFP surveillance*."

4. The authors mentioned expanding the ES sites that comes with related maintenance costs. But they haven't provided any cost-benefit analysis, for instance, if 54 ES-POP sites are enough to achieve a 11.3% national ES population coverage, will this implementation be cheaper than the alternatives or financially doable?p

Response 4: We thank the reviewer for the comment on lacking any cost-benefit analysis. I agree that comparison between different surveillance systems in terms of cost and benefit has been a recent growing interest, but it is challenging to establish the way for a fair comparison between the two, which is out of scope in our study. In addition, even though costs for 'start-up' and the process of 100 samples were reported in Tebbens et al.,[7] this cost did not include any non-laboratory related costs (e.g. salary and transportation costs for collecting wastewater samples by a grab method), which might vary largely depending on geographical ES site scale, even with the same number of specimen. It is also worth noting that it is difficult to translate our simulated number of ES-covered patches into the actual number of wastewater plants, which is a limitation of our study. Since the cost-benefit analysis is not performed, we deleted the sentence related to cost from the abstract.

Regarding the financial aspect, we want to limit our discussion to the early detection aspect of the ES, and we guess the financial feasibility depends on the context. Within the cost analysis by Tebbens et al.,[8] we can find the global trend to invest more in ES rather than AFP surveillance.

Action: We removed the cost-related sentence from the Abstract and revised the Abstract (page 2, lines 25-27)
"*Detecting the circulation of poliovirus in its early stages is paramount for swift public health action. Environmental surveillance (ES) has the potential to enhance early detection ability, but the impact of spatial arrangement of ES sites on early detection remains unclear. Here ~* "

5. The sensitivity analysis was conducted for the IMP-POP/ES-POP scenario only. Can you please clarify why this one is chosen?

Response 5: We thank the reviewer for giving the opportunity to clarify the point. This is because we consider IMP-POP/ES-POP scenario to be the most plausible scenario. Considering the latent period of polio patients (assumed to be 4 days), people can move for a long distance, which we think can be approximated

by a radiation model (supporting IMP-POP). Also, many ES sites in South Africa were concentrated in the most populous areas (supporting ES-POP).

6. Lines 209-211, the sensitivity analysis results seem natural, as if people travel frequently and more patches are used, the early detection rate will be higher. A more rigorous sensitivity analysis can be helpful to understand the effectiveness of ES, for example, using interaction of the factors (such as basic reproduction and travel rate) against the number of patches.

Response 6: We thank the reviewer for suggesting a more rigorous sensitivity analysis using an interaction of the factors. Although we understand the reviewer's concern about the non-linear behaviours of our model for specific parameter combinations, we opted not to investigate more on this aspect since importation scenarios and ES site layout have much larger impacts on ES effectiveness (Fig 2). Despite the difference in the magnitude of simulated early detection probabilities, the qualitative relationship between ES and early detection remains almost similar for the parameters included in Fig 3. This is in stark contrast to Fig 2, which shows the expected benefits from expanding ES sites were not linear for some scenarios.

7. Lines 497-498, if n_{i,t} is an index variable, then how can it be used to characterize the Binomial distribution(n, p), where n = the total number of samples takes. Please, clarify.

Response 7: We thank the reviewer for the question. Since we assume only one sample is collected from each site at time t, n_{i,t} takes only zero or one. Thus, it is possible to interpret the Binomial distribution as the Bernoulli distribution in our case.

Since we restructured the manuscript, we removed Equation 2 from our main manuscript and the same description can be found in Equation S6 in the supplementary material.

Action: We clarified this point in the supplementary (page 12, lines 268-270).
"*Let $n_{i,t}$ be the indicator variable for conducting environmental sampling at patch i at day t (e.g. given monthly sampling, $n_{i,t}$ takes one on a single day, and takes zero for the remaining days within a month in ES-covered patches and always takes zero in non-ES-covered patches),~*"

Reviewer #3: The manuscript demonstrates scientific merit but would benefit from improvements in structural organization, refinement of model assumptions regarding immunized individuals, and stronger efforts to link simulation patches to real-world geography.

1. The abstract is 276 words, which appears to be relatively long compared to typical journal standards. Consider reducing the length of the abstract to enhance conciseness and better align with common practices in academic journals.

Response 1: We thank for the reviewer's suggestion to shorten the abstract. Following the suggestion, we have shortened the abstract to 241 words.

Action: We have shortened the abstract (page 2, lines 25-42).
"*Detecting the circulation of poliovirus in its early stages is paramount for swift public health action. While Environmental surveillance (ES) is promising for enhancing early pathogen detection, the influence of spatial arrangement of ES sites on early detection remains unclear. Here, we aim to assess the early detection ability of ES by varying the number and location of ES sites using the simulation-based approach utilising geographic and demographic characteristics of South Africa as a case study of a non-endemic country. We developed a stochastic meta-population model among unimmunised children aged under 5 years old,*

assuming a single introduction of wild poliovirus serotype 1. We constructed six scenarios by combining three importation risk distributions (predicated on population size, approximations of international inbound travel volume and border crossing volume) with two ES site layout strategies (proportionate to population size or importation risks via land border crossings). We showed a modest number of strategically positioned ES sites can achieve a high early detection ability given assumed importation risks were geographically confined while dispersed importation risks reduced the effectiveness of ES. Our sensitivity analysis suggested that implementing the ES across large areas with low sampling frequency consistently resulted in a better early detection ability against various importation scenarios than implementing the ES in limited areas with high sampling frequency. Although we acknowledge the challenges of translating our simulated outcomes for real-world situations, our study has implications for deciding the scale and site selection of ES."

2. The introduction is well-written, demonstrating fluency and a clear articulation of the research problem, motivation, and support from existing literature. The structure is unconventional, with the Results section preceding the Materials and Methods section. While this structure is not strictly prohibited by PLOS ONE, it may reduce the clarity and logical flow of the manuscript.

Response 2: We thank the reviewer for their appreciation of our introduction and for the suggestion for reordering the section. Following that, we reordered the Method first, restructured method subsections and revised the Methods and Results to enhance clarity and readability. See the major change points to the response to Reviewer 1 point (1) and refer to detailed change points in the attached tracked version of the manuscript.

3. Authors assume that transmission is limited to children under 5, with individuals aged 5 and above being fully immunized. This overlooks the potential role of immunized individuals as asymptomatic carriers and vehicles for poliovirus transmission. Dont you think?

Response 3: We thank the reviewer for the comment. We agree that adults would play a role in contributing to transmission, particularly in polio-free countries where immune gap cohorts lack the opportunity of being immunised from natural infection. We are aware that Blake et al.[9] showed the significant contribution of older children and adults (R>1) to the polio outbreak in the Republic of Congo. However, since data for vaccination coverage for older children and adults in South Africa were unavailable and our main focus is not a precise evaluation of the current ES sites in South Africa, we believe our current approach is still valid to evaluate the relationship between the spatial arrangement of ES and early detection. We have already described the limitations regarding this point in the third limitation in the Discussion (page 23, lines 495-500).

4. The authors note that "it is difficult to relate a patch in our simulations to real-world geographical settings." However, while this is acknowledged, it is plausible to relate patches to administrative units, population density maps, or urban-rural divisions.

Response 4: We thank the reviewer for the comment. We have intended to mention that it is difficult to relate our "20km x 20km grid" patches to the complex geometry of the real-world wastewater catchment. Since our expressions were not clear, we revised the paragraph for more clarity.

Action: We clarified the paragraph as follows and also incorporated revisions for Response 5 (pages 20-21, lines 436-451).

"*Our sensitivity analysis showed a different choice of the patch-level ES population coverage (pc) resulted in large variations in simulated early detection probability even under the same national ES population*

coverage (Fig 4C), posing the challenge of translating our simulation results into the practical ES implementation strategy. This is because our current simplified patch assumption (i.e. 20 x 20km grid) fails to accurately represent the complex geometry of the real-world wastewater catchment, lacking one-to-one spatial correspondence. Moreover, our model's implication that choosing as small pc as possible achieves the highest early detection ability is unrealistic, which is likely attributable to the violation of homogenous mixing assumptions within a patch for a small value of pc. Considering those limitations, a current research gap for model assumptions is to identify areas where homogenous mixing is held, and in other words, to identify the fragmentation level of patches, as is historically pointed out by several authors [38–41]. One study of COVID-19 provided some insights into the homogenous mixing assumptions. This study used virus genome data with corresponding resident addresses in Dundee, Scotland, suggesting around a 5km radius circle was well mixed in terms of genetic distance [42]. In addition, more granular data for wastewater catchment area and its population coverage can help develop a more accurate mathematical model, for example using a two-layer network model [43]."

5. Results are robust, but the authors acknowledge challenges in translating simulated outcomes to real-world settings. Continue emphasizing the limitations and uncertainties associated with model-based simulations, while discussing avenues for future validation and refinement using empirical data.

Response 5: We thank the reviewer for the comment. We clarified the uncertainty associated with the model assumptions and improved the descriptions related to avenues for future data. See Response 4 for the revision.

References

1. Kalkowska DA, Franka R, Higgins J, Kovacs SD, Forbi JC, Wassilak SGF, et al. Modeling Poliovirus Transmission in Borno and Yobe, Northeast Nigeria. Risk Anal Off Publ Soc Risk Anal. 2021 Feb;41(2):289–302.

2. Kalkowska DA, Duintjer Tebbens RJ, Thompson KM. Modeling Strategies to Increase Population Immunity and Prevent Poliovirus Transmission in the High-Risk Area of Northwest Nigeria. J Infect Dis. 2014 Nov 1;210(suppl_1):S412–23.

3. McCarthy KA, Chabot-Couture G, Shuaib F. A spatial model of Wild Poliovirus Type 1 in Kano State, Nigeria: calibration and assessment of elimination probability. BMC Infect Dis. 2016 Sep 29;16(1):521.

4. Grassly NC, Fraser C, Wenger J, Deshpande JM, Sutter RW, Heymann DL, et al. New strategies for the elimination of polio from India. Science. 2006 Nov 17;314(5802):1150–3.

5. Fine PEM, Carneiro IAM. Transmissibility and persistence of oral polio vaccine viruses: Implications for the Global Poliomyelitis Eradication Initiative. Am J Epidemiol. 1999 Nov 15;150(10):1001–21.

6. Sallah K, Giorgi R, Bengtsson L, Lu X, Wetter E, Adrien P, et al. Mathematical models for predicting human mobility in the context of infectious disease spread: introducing the impedance model. Int J Health Geogr. 2017 Nov 22;16(1):42.

7. Duintjer Tebbens RJ, Zimmermann M, Pallansch M, Thompson KM. Insights from a systematic search for information on designs, costs, and effectiveness of poliovirus environmental surveillance systems. Food Environ Virol. 2017 Dec;9(4):361–82.

8. Duintjer Tebbens RJ, Diop OM, Pallansch MA, Oberste MS, Thompson KM. Characterising the costs of the Global Polio Laboratory Network: a survey-based analysis. BMJ Open. 2019 Jan;9(1):e023290.

9. Blake IM, Martin R, Goel A, Khetsuriani N, Everts J, Wolff C, et al. The role of older children and adults in wild poliovirus transmission. Proc Natl Acad Sci. 2014 Jul 22;111(29):10604–9.
